# Flagellin-elicited adaptive immunity suppresses flagellated microbiota and vaccinates against chronic inflammatory diseases

**DOI:** 10.1038/s41467-019-13538-y

**Published:** 2019-12-11

**Authors:** Hao Q. Tran, Ruth E. Ley, Andrew T. Gewirtz, Benoit Chassaing

**Affiliations:** 1 Center for Inflammation, Immunity and Infection, Institute for Biomedical Sciences, Georgia State University, Atlanta, GA USA; 2 Department of Microbiome Science, Max Planck Institute for Developmental Biology, Tübingen, Germany; 3 Neuroscience Institute, Georgia State University, Atlanta, GA USA; 4 INSERM, U1016, team “Mucosal microbiota in chronic inflammatory diseases”, Paris, France; 5 Université de Paris, Paris, France

**Keywords:** Antibodies, Microbiome, Obesity, Inflammatory bowel disease

## Abstract

Alterations in gut microbiota composition are associated with metabolic syndrome and chronic inflammatory diseases such as inflammatory bowel disease. One feature of inflammation-associated gut microbiotas is enrichment of motile bacteria, which can facilitate microbiota encroachment into the mucosa and activate pro-inflammatory gene expression. Here, we set out to investigate whether elicitation of mucosal anti-flagellin antibodies by direct administration of purified flagellin might serve as a general vaccine against subsequent development of chronic gut inflammation. We show, in mice, that repeated injection of flagellin elicits increases in fecal anti-flagellin IgA and alterations in microbiota composition, reduces fecal flagellin concentration, prevents microbiota encroachment, protects against IL-10 deficiency-induced colitis, and ameliorates diet-induced obesity. Flagellin’s impact on the microbiota is B-lymphocyte dependent and, in humans, obese subjects exhibit increased levels of fecal flagellin and reduced levels of fecal flagellin-specific IgA, relative to normal weight subjects. Thus, administration of flagellin, and perhaps other pathobiont antigens, may confer some protection against chronic inflammatory diseases.

## Abstract (web summary)

Gut microbiota alterations, including enrichment of flagellated bacteria, are associated with metabolic syndrome and chronic inflammatory diseases. Here, Tran et al. show, in mice, that elicitation of mucosal anti-flagellin antibodies protects against experimental colitis and ameliorates diet-induced obesity.

## Introduction

An array of chronic inflammatory diseases are associated with dysbiosis in the intestinal microbiota and a breakdown in the normally mutually beneficial host–microbiota relationship. Such diseases include inflammatory bowel diseases (IBD) and diseases characterized by low-grade inflammation, such as metabolic syndrome. Perturbations in the microbiota do not merely mark disease. Rather, that microbiota transplantation from IBD patients or colitic animals to germfree recipient mice results in intestinal inflammation argues that altered microbiota has a key role in driving inflammatory disease^1–5^. Although the specific alterations in microbiota composition associated with inflammation vary across study cohorts, common features of dysbiosis frequently include reduced species diversity and increased relative abundance of Proteobacteria^6,7^. Moreover, we previously reported that, whether induced by transient presence of a pathobiont, an innate immune deficiency, or the consumption of dietary emulsifiers (commonly used food additive with detergent-like properties), one common feature of inflammation-associated microbiotas is increased levels of flagellin, which can occur owing to changes in species composition and/or microbial gene expression^3,4,8–10^. The link between elevated microbiota flagellin levels and intestinal inflammation is thought to involve flagellin’s ability to activate pro-inflammatory gene expression via TLR5 and the NLRC4 inflammasome. In addition, as the structural component of flagella, elevated levels of flagellin might reflect enriched levels of motile bacteria that have high ability to penetrate the mucus layer that serves to protect the host against microbial onslaught.

In accord with the notion that a microbiota expressing high levels of flagellin pose danger to the host, flagellin is also a dominant target of adaptive mucosal immunity, particularly in Crohn’s disease^11^. Yet, like many disease-associated immune responses, adaptive immunity to flagellin likely plays an important role in keeping microbes in check. Specifically, the coating of gut bacteria by flagellin-specific IgA, which normally occurs in homeostasis, suppresses levels of flagellated bacteria and guards against microbiota encroachment, which is thought to play a role in promoting both IBD and metabolic syndrome^8,12–14^. Hence, we hypothesize that boosting levels of mucosal flagellin-specific IgA might help keep flagellated bacteria in check and, consequently protect against development of chronic gut inflammation. Herein, we describe that repeated systemic administration of purified flagellin elicits a robust anti-flagellin fecal IgA response that serves to reshape microbiota composition, reduces flagellin expression, and protects against experimental colitis and metabolic syndrome.

## Results

### Flagellin administrations elicits systemic and mucosal antibodies

**Fig. 1:**
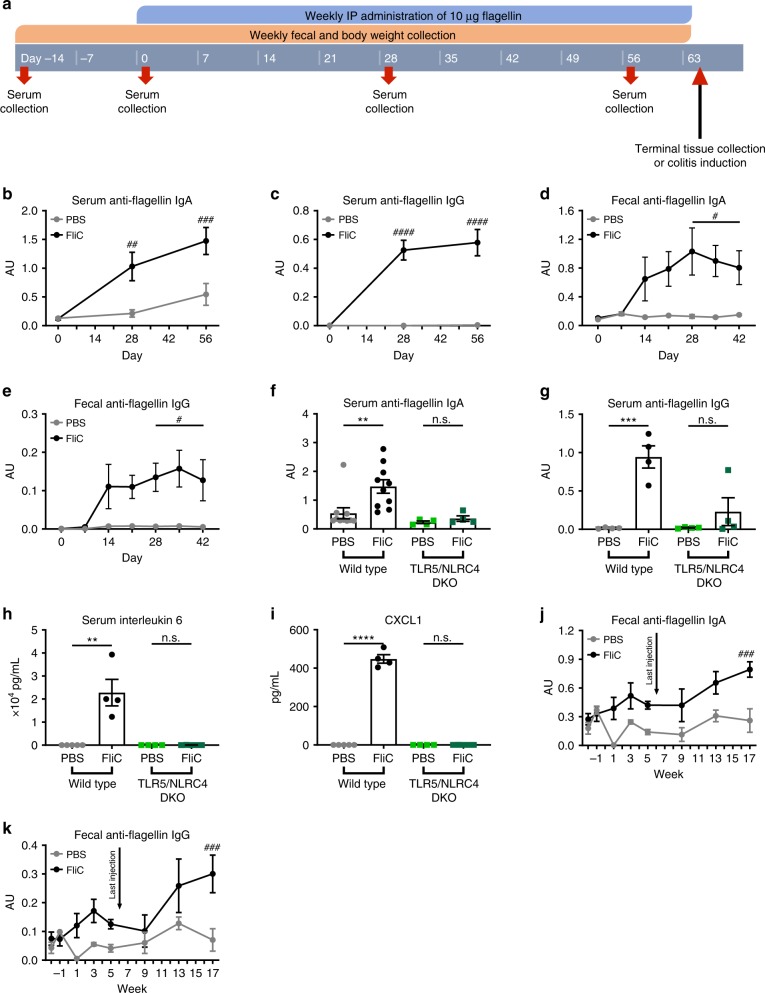
**Systemic flagellin administrations elicit systemic and mucosal antibodies to flagellin.** **a** 4-week old C57BL/6 J mice, wild type and TLR5/NLRC4 DKO, were purchased from The Jackson Laboratory and housed for 2 weeks before procedure in order to favor microbiota stabilization. Subsequently, flagellin (10 μg per mouse) was administered by intraperitoneal injections weekly for 9 weeks, whereas control mice received vehicle (PBS). Serum collection occurred on days −14, 0, 28, 56, and 63. Body weight measurements and fecal collection occurred prior to every flagellin administration. **b**–**c** Serum anti-flagellin IgA and IgG throughout the experiment, **d**–**e** fecal anti-flagellin IgA and IgG, **f**–**g** serum anti-flagellin IgA and IgG at day 56, **h** serum interleukin-6, and **i** CXCL1 at day 56 were analyzed using ELISA kits. **j**–**k** Fecal anti-flagellin IgA **j** and IgG **k** were also quantified up to 11 weeks after the final flagellin administration in mice receiving 6 weekly intraperitoneal injections of flagellin (10 μg per mouse). Data are the means ± S.E.M. Significance was determined using *t* test (**p ≤ 0.01 ***p ≤ 0.001 ****p ≤ 0.0001, n.s. indicates non-significant) or using one-way ANOVA corrected for multiple comparisons with a Bonferroni test (^#^*p* ≤ 0.05 ^##^*p* ≤ 0.01 ^###^*p* ≤ 0.001 ^####^*p* ≤ 0.0001, n.s. indicates non-significant). (*N*=4–5 mice from one out of three representative experiment). Source data are provided as a Source Data file.

The central hypothesis this study sought to test was whether an administration regimen of purified bacterial flagellin that elicited mucosal anti-flagellin antibodies might suppress levels of flagellated microbes and, consequently serve as a vaccine against chronic intestinal inflammation, which are associated with increased levels of flagellated pathobionts^3,6,9,10^. We selected *Salmonella*-derived flagellin (FliC) as a test immunogen, because our previous work indicated that it could elicit antibodies that recognized a range of flagellins from a variety of bacterial species, likely reflecting that some regions of the flagellin molecule are highly conserved^8,15^. Mice purchased from many commercial sources, such as Jackson Laboratories, which provided mice for this and our previous studies, exhibit very low baseline levels of anti-flagellin-antibodies and, accordingly, exhibit a typical primary response to initial immunization and a memory-type response to subsequent flagellin exposures^16^. A range of routes of administration were considered and tested. In accord with previous studies, orally administered flagellin failed to elicit a detectable immune response, likely reflecting its degradation by digestive tract proteases and/or inability to cross the mucosal epithelial surface that lines the gastrointestinal tract. Nasally administered flagellin, with or without cholera toxin, elicited detectable levels of fecal and serum flagellin-specific antibodies, but did so with high variability. Hence, as a means to test our hypothesis, we opted for repeated intraperitoneal injections of flagellin (10 weekly injections, at days 0, 7, 14, 21, 28, 35, 42, 49, 56 and 63) that resulted in robust levels of systemic (serum) and mucosal (fecal) anti-flagellin antibodies (Fig. 1a–e). In accord with our previous studies, such adaptive immune responses depended upon flagellin’s ability to activate innate immunity in that mice lacking both flagellin receptors, namely TLR5 and NLRC4, exhibited a near complete loss of flagellin-elicited antibodies^17^ (Fig. 1f, g). The high levels of anti-flagellin attained did not prevent injected flagellin from continuing to potently activate TLR5-mediated cytokine production (Fig. 1h, i). Fecal anti-flagellin antibodies induced by flagellin immunization were observed in the lumen in various regions of the gastrointestinal tract (duodenum, jejunum, ileum and colon, Supplementary Fig. 1). Anti-flagellin antibodies remained elevated 3 months after completion of the immunization regimen (latest time assayed), indicating a long-lasting response (Fig. 1j, k). Thus, we reasoned that IP injections served as a reasonable experimental model that could be used to explore how a high mucosal antibody response to flagellin might impact subsequent development of inflammatory disease.

### Flagellin administration alters the intestinal microbiota

**Fig. 2:**
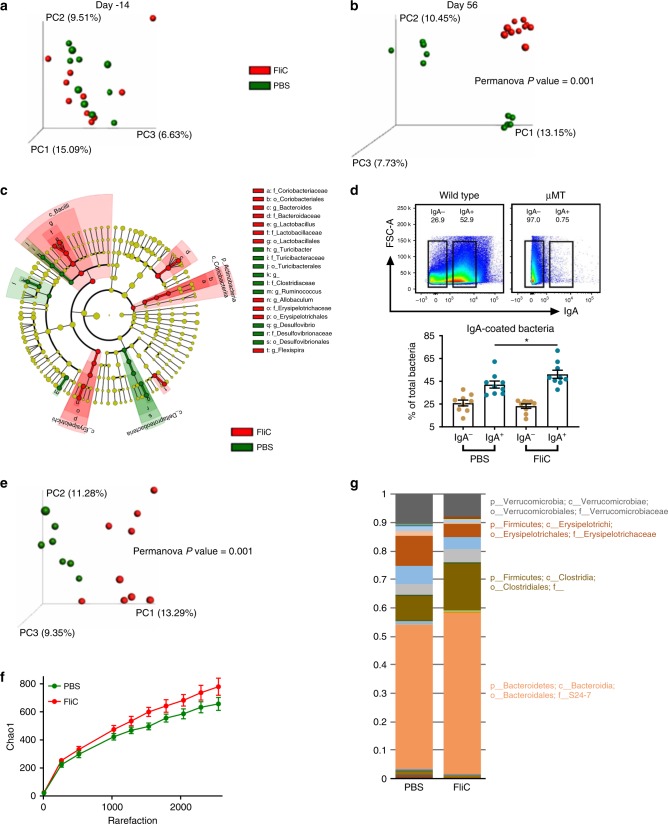
**Flagellin administration alters the intestinal microbiota towards a lower pro-inflammatory state.** 4-week old C57BL/6 J Wild Type mice were purchased from The Jackson Laboratory and housed for two weeks before procedure in order to favor microbiota stabilization. Subsequently, flagellin (10 μg per mouse) was administered by intraperitoneal injections weekly for 9 weeks, whereas control mice received vehicle (PBS). Fecal microbiota composition was analyzed using Illumina sequencing of the V4 region of 16 S rRNA genes. **a**–**b** Principal coordinates analysis (PCoA) of the unweighted UniFrac distance matrix at **a** day −14 and **b** day 56 (post stabilization, post immunization). **c** LEfSe analysis was performed in order to investigate microbiota taxa that were significantly altered by immunization at day 56 (post stabilization, post immunization), with green and red colors highlighting taxa significantly more abundant in PBS- and flagellin-treated mice, respectively. **d** Percentage of IgA^±^-coated bacteria in PBS- and FliC-treated mice, wherein the IgA^−^ and IgA^+^ gates were determined follows appropriate SSC-A/FSC-A gating of SytoBC^+^ cells in wild-type and μMT mice. **e** Principal coordinates analysis (PCoA) of the unweighted UniFrac distance matrix of IgA-coated bacteria. **f** Alpha diversity rarefaction using the Chao1 index of IgA-coated bacteria. **g** Taxa summarization of IgA-coated bacteria. In **a** and **b**, categories were compared and statistical significance of clustering were determined via Permanova. Data are the means ±S.E.M. Significance was determined using *t* test (*p ≤ 0.05). (*N*=4–5 mice from one out of three representative experiment). Source data are provided as a Source Data file.

We next analyzed the impact of our flagellin immunization regimen on the intestinal microbiota. We interrogated fecal microbiota composition by 16 S rRNA gene sequencing and analyzed the unweighted Unifrac distance, as previously described^3,18^. We utilized two cages of control and flagellin-immunized mice thus aiding our ability to distinguish any differences in composition owing to “cage-clustering” vs. treatment. As expected, such an approach found microbiota composition was not different based on treatment at day −14. However, flagellin immunization led to a strong alteration of microbiota composition in that both flagellin-treated cages of mice were markedly distinct from the PBS-treated mice at day 56 using principal coordinate analysis of the unweighted Unifrac distance (Fig. 2a, b). Moreover, the use of the weighted Unifrac distance still depicted distinct microbiota between PBS- and flagellin-treated mice, whereas cage clustering was much more subtle, as presented in Supplementary Fig. 2A, suggesting that microbiota members of low abundances are driving cage clustering in PBS-treated animals while more profound alterations are occurring in response to flagellin immunization. In accord with this notion, LEfSe analysis revealed that multiple OTUs are significantly increased or decreased in relative abundance in fecal microbiota derived from FliC-treated animals compared with PBS-treated animals despite the two cages of PBS-treated animals, exhibiting distinct microbiotas due to cage clustering (Fig. 2c and Supplementary Fig. 2B).

To better assess how FliC immunization impacted microbiota-IgA interactions, we next performed IgA-Seq on immunized and non-immunized animals using previously described flow sorting-based approaches^19,20^. This revealed that flagellin immunization is sufficient to significantly increase the proportion of fecal IgA-coated bacteria. Moreover, IgA immunization is inducing a significant shift in the IgA-coated bacterial population, with an increase in species richness and alteration in the proportion of numerous OTUs (Fig. 2d–g). Hence, these data suggest that anti-flagellin IgA induced by FliC immunization are binding to a variety of microbial members, in accord with our the notion that some immunogenic epitopes are shared between flagella derived from various species including Proteobacteria and Firmicute phyla^8^ and our earlier observation that immunization with *E. coli* flagellin-elicited antibodies that cross reacted with Clostridia flagellin^15^.

**Fig. 3:**
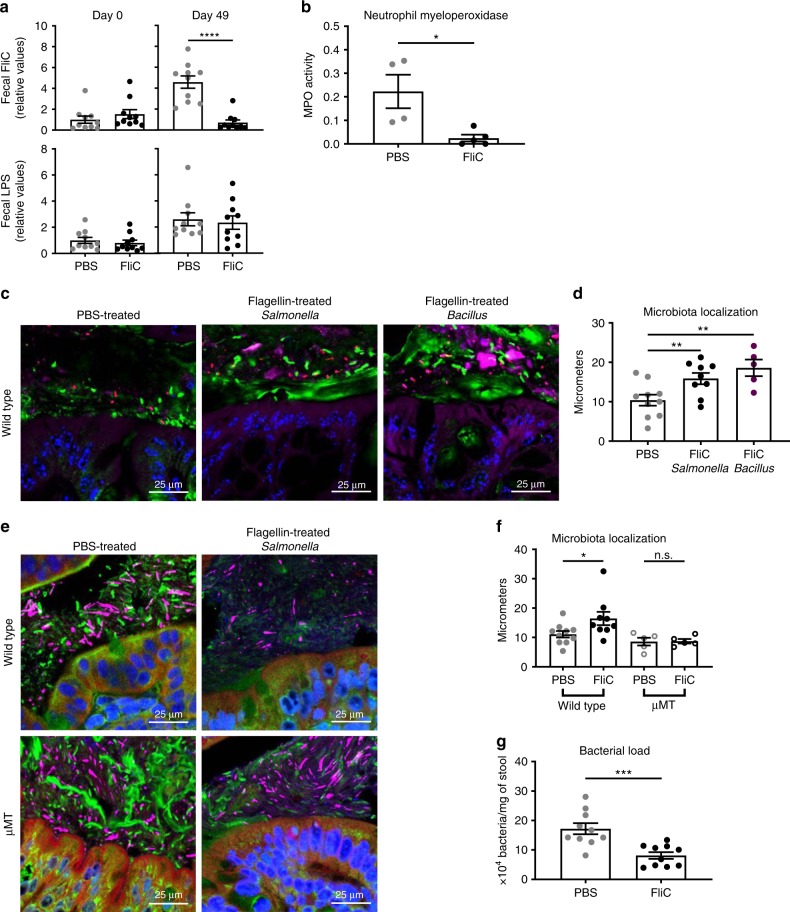
**Flagellin administration alters the intestinal microbiota toward a lower pro-inflammatory state.** **a** Fecal pro-inflammatory potential was analyzed using HEK 293 cells expressing mTLR5 or mTLR4 measuring bioactive flagellin and lipopolysaccharide, respectively. **b** Colonic myeloperoxidase quantification of 4-week old, wild-type C57BL/6 J mice after receiving either vehicle or 10 μg of flagellin by intraperitoneal injections weekly for 9 weeks. **c**–**f** Colonic microbiota localization analysis of wild type and μMT mice treated with PBS, *Salmonella*-derived flagellin, or *Bacillus*-derived flagellin. **c**, **e** Confocal microscopy analysis of colonic microbiota localization; Muc2 (green), actin (purple), bacteria (red), and DNA (blue). **d**, **f** Distances of closest bacteria to colonic intestinal epithelial cells (IEC) per condition over 2–3 high-powered fields per mouse. **g** Fecal bacterial load determined by qPCR analysis of 16 S bacterial DNA in the fecal contents of mice treated with PBS or flagellin. Data are the means ±S.E.M. Significance was determined using *t* test (*p ≤ 0.05 **p ≤ 0.01 ***p ≤ 0.001 ****p ≤ 0.0001, n.s. indicates non-significant). (*N*=4–5 mice from one out of three representative experiment). Source data are provided as a Source Data file.

We next sought to investigated the functional consequences of these alterations in microbiota composition in FliC-treated mice. First, we examined if the immunization lowered microbiota expression of level of flagellin, which might impact ability of microbiota to activate innate immune/pro-inflammatory gene expression. Using our previously described TLR5-expressing cell-based assay to quantify fecal bioactive flagellin^3,10^, we observed that flagellin immunization resulted in decreased fecal flagellin relative to PBS-treated age- and gender-matched control mice (Fig. 3a). In contrast, such immunization did not significantly impact levels of fecal LPS. FliC immunization also reduced level of flagellin in the colonic lumen but did not impact this parameter in the small intestine, possibly reflecting that such levels were already relatively low to begin with (Supplementary Fig. 1). Quantitation of fecal flagellin by western blotting confirmed results of the HEK-cells-based assay in showing that feces from flagellin-immunized mice indeed contained less flagellin than feces from non-immunized mice (Supplementary Fig. 3A).

Reduced levels of fecal/colonic flagellin suggest a less-motile microbiota with less potential to encroach upon host epithelial cells and initiate intestinal inflammation. In accord, we observed decreased myeloperoxidase activity following FliC immunization (Fig. 3b). To examine if this might reflect decreased microbiota encroachment, we next examined microbiota localization via confocal microscopy. In PBS-treated animals, the average closest bacteria per high-powered field microbiota were localized at 10.38 ±(S.E.M.) 1.40 μm from the epithelium, whereas in *Salmonella*-derived FliC-treated animals, such closest microbiota were located 15.88 μm ± 1.43 μm from the epithelium (*P* < 0.05, using *t* test) (Fig. 3c, d). Moreover, quantification of fecal bacterial loads demonstrated a significant decrease in immunized mice compared with controls (Fig. 3g), suggesting broad impact of flagellin immunization on the microbiota in terms of biomass, composition, localization, and pro-inflammatory potential. Importantly, the ability of flagellin immunization to impact microbiota was not specific to *Salmonella* FliC. Rather, immunizing mice with flagellin purified from *Bacillus subtilis* also increased fecal anti-flagellin IgA, as well as prevented microbiota encroachment and decreased microbiota pro-inflammatory potential (Supplementary Fig. 3B-D and Fig. 3d), suggesting that both pathogen- or commensal-derived flagellin are efficient in beneficially impacting the intestinal microbiota, again in accord with the notion that some regions of the flagellin molecules are conserved.

Although one can envisage a range of potential mechanisms whereby flagellin administration might impact the microbiota, we hypothesized that the flagellin-induced change in microbiota composition, flagellin levels, and localization observed here are the result of mucosal anti-flagellin antibodies. To test this notion, we next examined the extent to which mice unable to produce antibodies owing to their lack of mature B cells, namely μMT mice, would also exhibit an increased microbiota/epithelial cells distance following immunization. Importantly, in μMT mice, flagellin immunization regimen no longer resulted in an increase in bacterial–epithelial distance (Fig. 3e, f), thus arguing that a significant portion of flagellin’s impact upon the microbiota is mediated by anti-flagellin antibodies.

### Flagellin administration protects against colitis

**Fig. 4:**
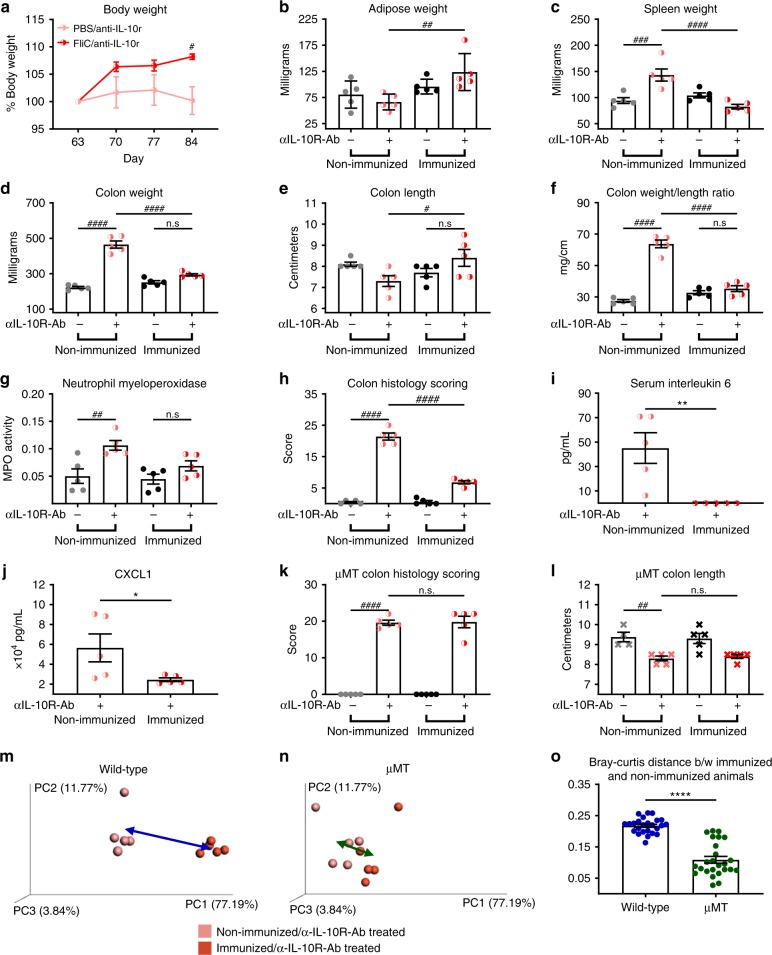
**Flagellin administrations protect against immune dysregulation-induced colitis.** 4–8-week old C57BL/6 J wild-type and μMT mice received either vehicle or 10 μg of flagellin by intraperitoneal injections weekly for 9 weeks. Subsequently, animals were treated weekly for 4 weeks by 1 mg of anti-IL-10R antibody intraperitoneally to induce intestinal inflammation. Biometric data of Wild Type animals represented by **a** body weight, **b** adipose weight, **c** spleen weight, **d** colon weight, **e** colon length, **f** and colon weight/length ratio. **g** Colonic myeloperoxidase levels. **h** Colon pathohistological scoring. **i**–**j** Serum interleukin-6 and CXCL1 following anti-IL-10R antibody regimen. Severity of colitis in μMT animal represented by **k** colon pathohistological scoring and **l** colon length. **m**–**o** Principal coordinate analysis of the Bray–Curtis distance using a matrix containing all the morphometric and molecular parameters presented in **a**–**l**. Data are the means ± S.E.M. Significance was determined using *t* test (*p ≤ 0.05 **p ≤ 0.01 ****p ≤ 0.0001) or using one-way ANOVA corrected for multiple comparisons with a Bonferroni test (#*p* ≤ 0.05 ^##^*p* ≤ 0.01 ^###^*p* ≤ 0.001 ^####^*p* ≤ 0.0001, n.s. indicates non-significant). (*N*=4–5 mice from one out of two representative experiment). Source data are provided as a Source Data file.

Flagellin is reported to be a dominant antigenic driver of Crohn’s disease^11^, whereas microbiota encroachment is a feature of IBD in general^12,21^. Hence, we hypothesized that the above-described immunization regimen, which decreased levels of flagellin and increased bacterial–epithelial distance, might protect mice against colitis. To examine this possibility, we subjected flagellin-immunized and control (PBS-treated) mice to immune dysregulation-induced colitis, which was achieved by weekly injections of a IL-10 receptor-neutralizing antibody. In accord with previous work, such blockade of IL-10 signaling resulted in typical features of colitis, including loss of weight/adiposity, splenomegaly, colomegaly, colon shortening, elevated MPO, increase in pathohistological scoring, and elevations in serum IL-6 and CXCL1 (Fig. 4). Importantly, all of these parameters were reduced in flagellin-immunized mice, indicating that flagellin immunization had potential to protect against colitis (Fig. 4a-j and Supplementary Fig. 4A). To determine whether such protection was indeed dependent on flagellin-elicited antibodies, the experiment was subsequently repeated using μMT mice. Such mice exhibited indices of colitis, such as colon shortening, which was not prevented by flagellin immunization, suggesting that flagellin-elicited antibodies contribute to colitis protection (Fig. 4k, l and Supplementary Fig. 4B). Next, multivariate test analysis using all the above-described measures (Fig. 4a–l) was used to measure the impact of flagellin immunization on intestinal inflammation. Bray–Curtis distance, presented Fig. 4m–o, demonstrate that flagellin immunization is having a significant impact on WT animals in protecting them against intestinal inflammation, whereas the protective effects are very modest in μMT mice, as highlighted by a smaller Bray–Curtis distance between immunized and non-immunized animals compared with WT animals (Fig. 4o).

**Fig. 5:**
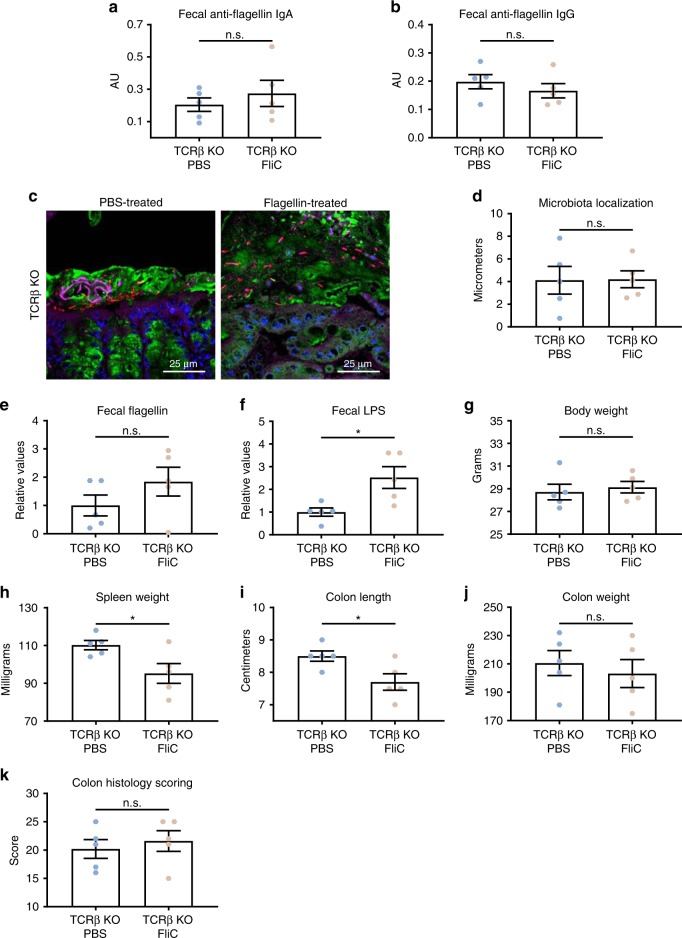
**Beneficial effects of flagellin immunization are abolished in TCRβ KO mice.** 4-week old C57BL/6 J TCRβ KO mice were purchased from The Jackson Laboratory and housed for 2 weeks before procedure in order to favor microbiota stabilization. Next, flagellin (10 μg per mouse) was administered by intraperitoneal injections weekly for 9 weeks, whereas control mice received vehicle (PBS). Subsequently, animals were treated weekly for 4 weeks by 1 mg of anti-IL-10R antibody intraperitoneally to induce intestinal inflammation. **a**–**b** Fecal anti-flagellin IgA and IgG quantified using ELISA. **c** Confocal microscopy analysis of colonic microbiota localization; Muc2 (green), actin (purple), bacteria (red), and DNA (blue). **d** Distances of closest bacteria to colonic intestinal epithelial cells (IEC) per condition over 2–3 high-powered fields per mouse. **e**–**f** Fecal flagellin and LPS quantified using HEK 293 cells expressing mTLR5 or mTLR4. **g** Body weight, **h** spleen weight, **i** colon length, **j** colon weight, and **k** colon pathohistological scoring. Data are the means ±S.E.M. Significance was determined using *t* test (*p ≤ 0.05, n.s. indicates non-significant). (*N*=4–5). Source data are provided as a Source Data file.

Generation of serum antibodies to flagellin are absolutely dependent upon CD4 T-cell helper in that such antibodies are not generated in TCRβ KO mice^15^. Analogously, repeated FliC immunizations in these mice did not elicit increases in fecal anti-flagellin IgA or IgG (Fig. 5a, b). Importantly, such FliC immunization of TCRβ KO mice did not reduce microbiota encroachment nor protected against colitis in mice subjected to IL-10 receptor neutralization, further supporting the need for the adaptive immune system to mediate beneficial effects of flagellin FliC immunization (Fig. 5c–k).

**Fig. 6:**
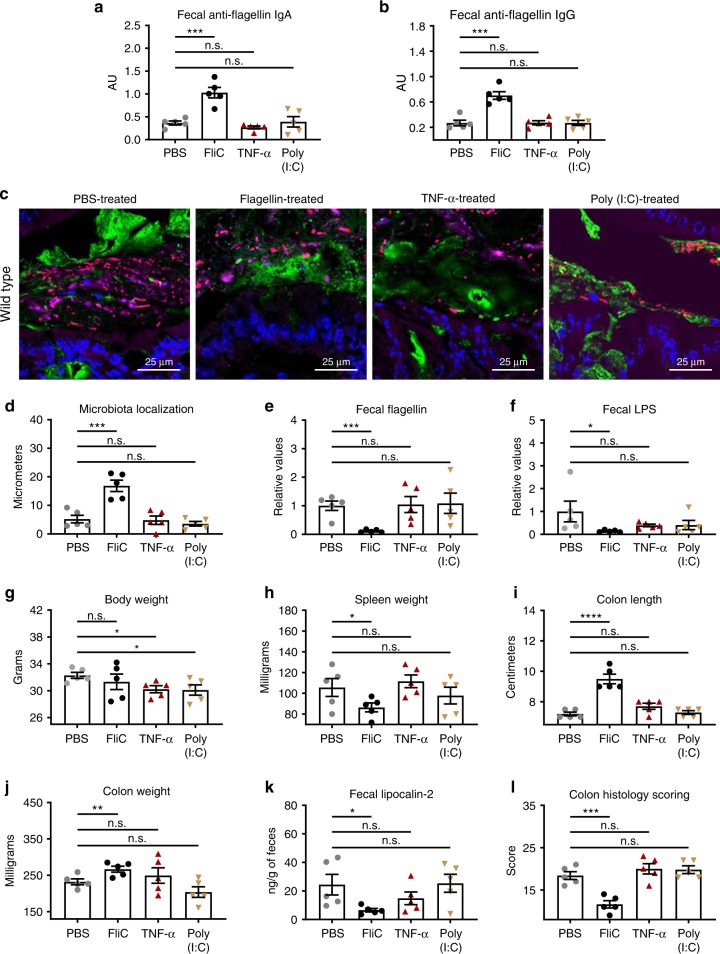
**Specificity of the beneficial effects induced by flagellin immunization.** 4-week old C57BL/6 J, wild-type mice, were purchased from The Jackson Laboratory and housed for 2 weeks before procedure in order to favor microbiota stabilization. Subsequently, mice were treated with either flagellin (10 μg per mouse), TNF-α (50 μg/kg body weight), or Poly (I:C) (10 μg/kg body weight) via intraperitoneal injections weekly for 9 weeks, whereas control mice received vehicle (PBS). Subsequently, animals were treated weekly for 4 weeks by 1 mg of anti-IL-10R antibody intraperitoneally to induce intestinal inflammation. **a**–**b** Fecal anti-flagellin IgA and IgG quantified using ELISA. **c** Confocal microscopy analysis of colonic microbiota localization; Muc2 (green), actin (purple), bacteria (red), and DNA (blue). **d** Distances of closest bacteria to colonic intestinal epithelial cells (IEC) per condition over 2–3 high-powered fields per mouse. **e**–**f** Fecal flagellin and LPS quantified using HEK 293 cells expressing mTLR5 or mTLR4. **g** Body weight, **h** spleen weight, **i** colon length, and **j** colon weight. Colitis severity was assessed by **k** fecal lipocalin-2 concentration and **l** colon pathohistological scoring. Data are the means ±S.E.M. Significance was determined using *t* test (*p ≤ 0.05 **p ≤ 0.01 ***p ≤ 0.001 ****p ≤ 0.0001, n.s. indicates non-significant). (*N*=4–5 mice). Source data are provided as a Source Data file.

To further explore, in WT mice, the specificity of the protection observed with flagellin immunization, we also examined if other inflammatory stimuli could potentially protect against intestinal inflammation. We repeatedly treated mice with flagellin, Poly (I:C), or TNF-α, the latter aiming to mimic innate immune response induced by flagellin injection. Following such treatment, colitis was induced by anti-IL-10R treatment. As expected, among these agonists, only flagellin immunization elicited flagellin-specific antibodies (Fig. 6a, b). Furthermore, only flagellin protected against microbiota encroachment (Fig. 6c, d), decreased microbiota pro-inflammatory potential (Fig. 6e, f) and prevented colitis, as revealed by decreased spleen weight, increase in colon length and weight, decrease in fecal level of lipocalin-2 and decrease in colonic histopathological score (Fig. 6g–l and Supplementary Fig. 5). Altogether, these data support the notion that, in the model system used herein, protection against intestinal inflammation in FliC-immunized mice is mediated by elicitation of an adaptive immune response to bacterial flagellin.

Another experimental approach to achieving loss of IL-10 function, thus making mice prone to developing microbiota-dependent colitis, is the use of mice lacking the IL-10 gene. Hence, we next immunized IL-10 KO mice subjected to flagellin treatment weekly from 4–14 weeks of age, and thereafter, examined the extent to which such flagellin immunization impacted their development of colitis. In accord with previous observations that IL-10 KO mice make robust, somewhat exaggerated immune responses, we observed that our immunization regimen not only induced robust fecal anti-flagellin IgA, but IgG as well (Supplementary Fig. 6A, B). Although the control (PBS-treated) IL-10 KO mice used here did not develop severe colitis, they did display short colons, and low but detectable level of colon MPO activity, both suggestive of chronic inflammation, which was attenuated by flagellin immunization (Supplementary Fig. 6C–I). These results support the notion that immunization with flagellin can protect against immune dysregulation-induced colitis, in which the concentration of fecal and serum anti-flagellin IgA positively correlate with protection against chronic intestinal inflammation (Supplementary Fig. 6J–L). Interestingly, flagellin immunization, despite inducing a strong decrease in fecal flagellin expression and a reproducible protection against IL-10 receptor neutralization-induced colitis, was not sufficient to protect mice against DSS colitis, which does not require immune dysregulation nor depend upon the presence of a microbiota^22^ (Supplementary Fig. 7).

### Flagellin immunization protected against diet-induced obesity

**Fig. 7:**
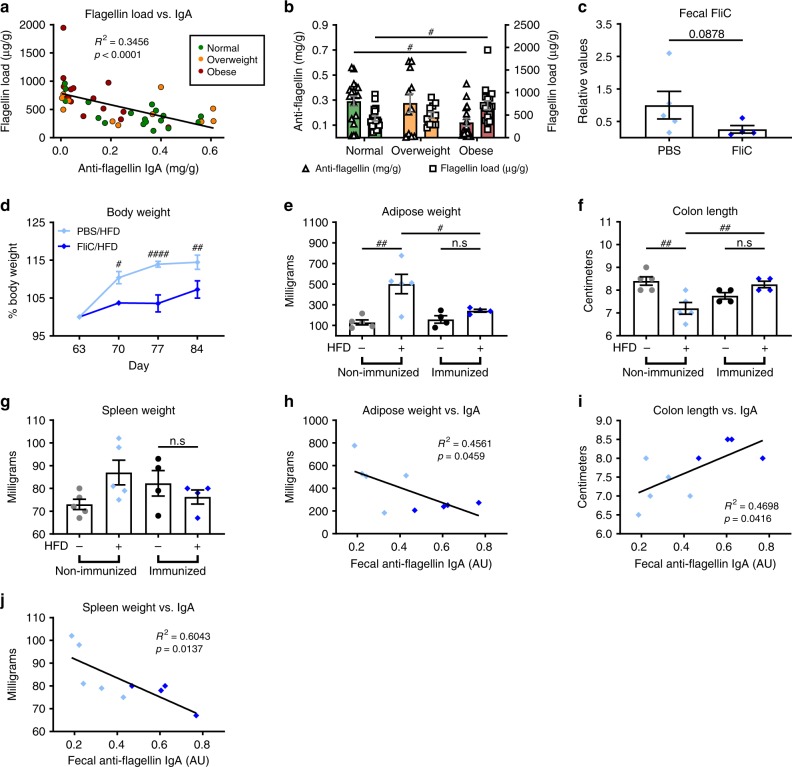
**Flagellin immunization protected against high-fat diet-induced obesity.** **a** Flagellin load (*y* axis) inversely correlate with anti-flagellin IgA concentration (*x* axis) in humans. R2 represents the coefficient of determination. **b** Mean concentration ± S.E.M. of flagellin load and anti-flagellin IgA concentration for human subjects segregated by their BMI to normal (18.5–24.9, *N* = 17), overweight (25–29.9, *N* = 11) or obese (> 30, *N* = 15). Flagellin concentration is denoted on the left *y* axis and anti-flagellin IgA concentration is denoted on the right *y* axis. ^#^*P* < 0.05; one-way analysis of variance (ANOVA). **c**–**j** 8-week old, C57BL/6 J mice were purchased from The Jackson Laboratory and housed for two weeks before procedure in order to favor microbiota stabilization. Subsequently, flagellin (10 μg per mouse) was administered by intraperitoneal injections weekly for 9 weeks, whereas control mice received vehicle (PBS). Subsequently, animals were treated with high-fat diet (60% kcal from fat) for 4 weeks. **c** Fecal flagellin quantified using HEK 293 cells expressing mTLR5. **d** Body weights were measured weekly and expressed as relative values, day 63 (post immunization, pre high-fat diet treatment) being define as 100%. **e** Adipose weight. **f** Colon length. **g** Spleen weight. **h**–**j** Day 63 fecal anti-flagellin IgA correlated with adipose and spleen weights, as well as, colon length. Data are the means ± S.E.M. Significance was determined using linear regression analysis (for **h**, *p* values shown), *t* test (for **c**), or one-way ANOVA corrected for multiple comparisons with a Bonferroni test (^#^*p* ≤ 0.05 ^##^*p* ≤ 0.01 ^####^*p* ≤ 0.0001, n.s. indicates non-significant) for **e**–**g**. (*N*=4–5 mice from one out of two representative experiment). Source data are provided as a Source Data file.

Recent work by ourselves and others have shown that microbiota encroachment is not only associated with IBD but also is a feature of metabolic syndrome in humans^14^. We suspected that one factor that might contribute to such microbiota encroachment is a relatively low amount of flagellin-specific mucosal IgA relative to the amount of flagellated bacteria present. To examine this possibility, we measured the levels of flagellin and flagellin-specific IgA in a collection of fecal samples previously isolated from subjects ranging in BMI from healthy to overweight and obese^23^. Consistent with our observations in mice, we found that anti-flagellin IgA levels were inversely proportional to flagellin in human feces (*R*^2^= 0.3456, *P* < 0.0001, Fig. 7a). We observed that levels of fecal flagellin were higher in overweight persons, relative to normal weight subjects, and further significantly increased in obese persons. In contrast, levels of flagellin-specific IgA were reduced in fecal samples derived from obese subjects compared with lean subjects (Fig. 7b). These results are in accord with the possibility that insufficient immune responses to flagellin contribute to low-grade inflammation thought to promote this disorder and, consequently, that immunization with flagellin may be able to prevent or ameliorate this disorder. To examine this possibility, flagellin-immunized and control PBS-treated mice were monitored following administration of an obesogenic high-fat diet (HFD). We observed that flagellin-immunized mice gained less weight and exhibited less adiposity compared with non-immunized animals (Fig. 7c–e). Moreover, flagellin immunization was associated with decreased intestinal inflammation, as revealed by a protection against colon shortening and spleen enlargement that normally result from HFD treatment (Fig. 7f, g). Correlation analysis revealed that fecal anti-flagellin IgA load correlate with the degree of protection observed against adiposity and inflammation (Fig. 7h–j). Altogether, these results suggest that immunization can protect mice against low-grade inflammation and increased adiposity associated with a HFD regimen.

## Discussion

An array of chronic inflammatory diseases, including IBD and metabolic syndrome, are associated with alterations in gut microbiota composition^24–27^. That transplants of these disease-associated microbiotas can recapitulate many disease features in recipient mice indicates that such alterations play a role in driving these inflammatory diseases^1,28,29^. Although, across different studies, there is high variance in the specific bacterial species whose enrichment and/or depletion is associated with disease, one general microbiota feature, common to numerous sequence-based studies in mice and humans, is enrichment of flagellated bacteria. Increases in flagellated bacteria can result from increases in Gamma-Proteobacteria, including motile pathobiont *Escherichia coli* strains^6,7^, but can also result from other classes of bacteria, especially Firmicutes, upregulating motility-related gene expression^8^. In accord, functional-based microbiotas assessments reveal that these disease-associated microbiotas express high levels of flagellin and, concomitantly, have a high capacity to penetrate the mucus layer of their host^3,4,9^. We hypothesize that these encroaching bacteria play an outsize role in inducing the pro-inflammatory gene expression characteristic of gut inflammation. Although the intestine possesses a number of innate immune mechanisms to keep the inner mucus layer free of bacteria, several observations suggest that adaptive immunity, in particular mucosal production of flagellin-specific IgA, also plays a key role in keeping motile bacteria in check. For example, in mice, absence of flagellin-specific IgA, owing to absence of TLR5-mediated promotion of adaptive immunity, resulted in increased levels of flagellin and microbiota encroachment^8^. Moreover, as shown herein, relative to healthy humans, overweight and obese persons exhibited decreased levels of flagellin-specific IgA and increased levels of fecal flagellin. Importantly, recent studies revealed that obese mice have a decreased number of IgA+ immune cells, whereas HFD-fed IgA-deficient mice present altered glucose metabolism^30^ and mice deficient in intestinal IgA production present metabolic deregulations^31^, further highlighting the importance of IgA in protecting against chronic gut inflammation.

These results support the notion that natural acquisition of flagellin-specific IgA protects against chronic inflammatory diseases, thus leading to the suggestion that eliciting anti-flagellin antibodies via immunization might be a means to vaccinate against such diseases. Although some antibodies to bacterial flagella are highly species-specific, thus providing the basis for H-serotyping that can distinguish even closely related species of *E. coli*, many antibodies to flagellin recognize highly conserved flagellin epitopes, such that inoculation of mice with *E. coli* flagellin generated antibodies that exhibited considerable cross reactivity with a *Clostridia* flagellin^15^, thought to be one target of the increased antibody response to flagellin^11^. Thus, even though the specific bacteria that might encroach upon the epithelium in various disease states have not been identified, and likely vary considerably in different hosts and disease states, we hypothesized that eliciting a robust anti-flagellin response might nonetheless provide a degree of protection across a range of chronic inflammatory diseases. Accordingly, as shown herein, elicitation of flagellin-specific IgA via exogenous administration of flagellin has potential to protect against some chronic inflammatory diseases, including colitis and obesity. Although the regimen used here of 10 injections of flagellin might not be practical in humans, the observation that almost all humans exhibit considerable basal antibody responses to flagellin leads us to speculate that humans might exhibit a memory-type response to initial flagellin treatment, which make the approach more effective in humans than in mice and/or enable fewer injections^32,33^.

Adaptive immunity to flagellin is an established feature of IBD, especially Crohn’s disease, in that IBD patients have elevations in serum antibodies (IgG and IgA) and a greater frequency of flagellin-specific CD4 T cells^11,34–36^. That transfer of flagellin-specific T cells to immune deficient mice can induce colitis demonstrates potential for flagellin-specific immunity to be detrimental, and underscores that caution should be taken in the deliberate elicitation of flagellin-specific immunity^11^. Yet, whether flagellin-targeted immune responses actually drive IBD in humans or are a beneficial adaptation that protects IBD patients from their microbiota is not known, and may in fact vary substantially depending upon the time such responses are elicited together with the underlying cause of disease. For example, it seems reasonable to envisage that persons with an innate immune deficiency, for example, those with non-functional Nod2, might benefit from deliberate induction of adaptive immunity to flagellin, whereas such treatment might be detrimental to those with inherent immune dysregulation, such as persons with mutations in IL-23 and/or IL-10 signaling pathways. However, that administration of flagellin to IL-10 KO mice elicited exaggerated antibody responses to flagellin (relative to WT mice) and reduced subsequent development of colitis suggests that even hosts with inherent immune dysregulation can benefit from elevated immune responses to flagellin, at least if elicited prior to disease development. Another possibility is that flagellin-specific antibodies are beneficial, perhaps in part because they can exclude flagellated bacteria from the inner mucus and/or by enchaining growing bacteria^37^, whereas activation of flagellin-specific T cells, which might occur as result of altered gut permeability, can drive detrimental immune activation. At last, given that we note that immune responses to flagellin are normally tightly compartmentalized to the mucosal compartment^38^, it is possible that mucosal responses to flagellin are beneficial while systemic responses are detrimental. Although the systemic administration used here elicited a potent systemic response, we envisage that development of approaches to administer flagellin to the gut mucosal immune system, perhaps with nanoparticles specifically targeting the mucosa, might provide a means to preferentially elicit mucosal antibodies, which may prove safer and more effective.

Although numerous remaining questions highlight the need for extensive further pre-clinical development of using flagellin administration to vaccinate against colitis, we nonetheless submit that our results herein demonstrate the potential for this approach to prevent gut inflammation, whereas future experiments will investigate the potential of such an approach to cure established disease. Indeed, should elicitation of flagellin-specific mucosal antibodies keep motile bacteria in check, prevent microbiota encroachment, and result in a generally less pro-inflammatory microbiota in humans, we submit this approach may reduce development of a broad array of inflammatory diseases including IBD, metabolic syndrome, and perhaps colon cancer. Indeed, we note that bacteria that have been proposed to promote colon cancer, including *E. coli* and *B. fragilis*, are often flagellated. Thus, caveats notwithstanding, we submit that administration of flagellin has potential to vaccinate against an array of diseases associated with, and driven by, gut inflammation.

## Methods

### Mice and flagellin immunization

Monomeric flagellin was purified from flagella isolated from *Salmonella* Typhimurium (SL3201, *fljB*^−^) via HPLC, and purity was verified as previously described^39,40^ . C57BL/6 mice (WT, TLR5/NLRC4 DKO^41^, μMT^42^, IL-10 KO^43^, TCRβ KO^44^ were maintained at Georgia State University, Atlanta, Georgia, USA under institutionally approved protocols (IACUC # A14033 and A18006). Mice were immunized with either *Salmonella* Typhimurium-derived flagellin (10 μg; described above), *B. subtilis*-derived flagellin (10 μg; InvivoGen, tlrl-bsfla), TNF-α (50 μg/kg body weight; R&D Systems, 410-MT-050) or Poly (I:C) (10 μg/kg body weight; Sigma, P1530) through intraperitoneal injections weekly for a total of 10 injections, with control mice being administered vehicle (PBS). For experiments utilizing anti-IL-10R antibody, 1 mg of anti-IL-10R (Bio X Cell, BE0050) antibody was administered through intraperitoneal injections following flagellin immunization weekly for 4 weeks. For DIO model, mice were fed with a 60% kcal from fat diet (Research Diet, D12492) for 4 weeks. Mice were littermates and group-housed. Mice were killed by CO_2_ inhalation, and colon length, colon weight, spleen weight and adipose weight were measure. Serum, feces, organs, as well as intestinal contents from the duodenum, ileum, and jejunum were collected for downstream analysis.

### Fecal sample preparation for immunoglobulin quantification

Fecal sample collection from mice occurred up to 3 months after the final flagellin administration. Sample preparation for ELISA has been previously described^45^. In brief, 100 mg of fecal pellets were homogenized in 3 mL of collection media consisting of 0.05 mg soybean trypsin inhibitor per ml of a 3:1 mixture of 1× PBS and 0.1 m EDTA, pH 7.4. Following centrifugation at 1800 rpm for 10 min, the supernatant was centrifuged again at 14,000 rpm for 15 min at 4 °C, and final supernatant was collected and stored with 20% glycerol and 2 mm phenylmethylsulfonyl fluoride (Sigma, P-7626) at − 20 °C until analysis.

### Fecal and serum anti-flagellin IgA/IgG

Quantification of anti-flagellin- specific IgA and IgG has been previously described^32–34^. In brief, 96-well microtiter plates (Costar, Corning, New York) were coated with 100 ng/well of either laboratory-made *Salmonella* Typhimurium-derived or purchased *B. subtilis*-derived flagellin in 9.6 pH bicarbonate buffer overnight at 4 °C. Serum or fecal samples from mice were then applied either pure or at a 1:100 dilution for 1 h at 37 °C. After incubation and washing, the wells were incubated with either horseradish peroxidase-linked anti-mouse IgG (GE Healthcare Life Sciences, NA931V) or horseradish peroxidase-linked anti-mouse IgA (Southern Biotech, 1040–05). Quantification of immunoglobulin was then developed by the addition of 3,3′,5,5′-Tetramethylbenzidine and the optical density was calculated by the difference between readings at 450 nm and 540 nm.

### Fecal lipocalin-2 quantification

As previously described^46^, frozen fecal samples were reconstituted in PBS containing 0.1% Tween 20 at 100 mg/ml and vortexed for 20 min The homogenate was then centrifuged at 12,000 rpm for 10 min at 4 °C. Clear supernatants were collected and stored at − 20 °C until analysis. Lcn2 levels were measured in the supernatants using DuoSet Murine Lcn2 ELISA kit (R&D Systems, DY1857).

### Myeloperoxidase quantification

Tissue samples were homogenized in 100 mg/mL of 0.5% hexadecyltrimethylammonium bromide (Sigma, H6269) in 50 mm PBS, pH 6.0, as previously described^46^. Following three cycles of freeze–thaw at − 80 °C and 37 °C, samples were sonicated and centrifuged at 14,000 rpm for 15 min at 4 °C. Supernatants were stored at − 20 °C until analysis. MPO was assayed in the supernatant by adding 1 mg/mL of dianisidine dihydrochloride (Sigma, D3252) and 5 × 10^−4^% H_2_O_2_ and the change in optical density measured at 450 nm.

### Serum CXCL1 and IL-6 quantification

Serum chemokine (C-X-C motif) ligand 1 (CXCL1) and Interleukin-6 (IL-6) concentrations were determined using Duoset cytokine ELISA kits (R&D Systems, DY453 and DY406, respectively) according to manufacturer’s instructions^46^.

### Fecal flagellin and lipopolysaccharide (LPS) load quantification

Quantification of flagellin and lipopolysaccharide was previously described using human embryonic kidney (HEK)-Blue-mTLR5 and HEK-Blue-mTLR4 cells, respectively (Invivogen, hkb-mtlr5 and hkb-mtlr4, respectively)^3,4^. Fecal material were resuspended in PBS to a final concentration of 100 mg/mL and homogenized for 10 s using a Mini-Beadbeater-24 without the addition of beads to avoid bacteria disruption. Samples were then centrifuged at 8000 × *g* for 2 min, serially diluted the resulting supernatant, and applied to mammalian cells. Purified *E. coli* flagellin and LPS (Sigma, L2887) were used for standard curve determination using HEK-Blue-mTLR5 and HEK-Blue-mTLR4 cells, respectively. After 24 h of stimulation, cell culture supernatants were applied to QUANTI-Blue medium (Invivogen, rep-qb1) and measured alkaline phosphatase activity at 620 nm after 30 min

### Western blot

Western blotting was performed as previously described^8,47^. In brief, feces were homogenized in PBS to a final concentration of 100 mg/mL, supernatant was collected following centrifugation at 12,000 rpm for 10 min at 4 °C. Fecal samples treated with Laemmli Sample Buffer (Bio-Rad, 161–0737) and β-mercaptoethanol (Bio-Rad, 161–0710) were resolved on polyacrylamide gels with Precision Plus Protein Standards (Bio-Rad, 161–0375) and transferred to nitrocellulose membranes. Membranes were then probed with an anti-flagellin antibody (dilution 1:200; Invivogen, mabg-flast). After washes, membranes were incubated with anti-mouse IgG-HRP conjugated secondary antibody (dilution 1:5000; GE Healthcare Biosciences, NA931V), and blots were detected using the ECL Western Blotting Detection (GE Healthcare Biosciences, RPN2106) on a Biorad Chemidoc machine.

### Bacterial load quantification

For quantification of fecal bacterial load, total fecal DNA was extracted from PBS- and flagellin-treated mice using QIAamp Fast DNA Stool Mini Kit (Qiagen, 51604). Purified DNA was subjected to qPCR using Qiagen QuantiFast SYBR Green PCR Kit (Qiagen, 204054) and 16 S ribosomal primers 515 F (5′-GGACTACHVGGGTWTCTAAT-3′) and 806 R (5′-GTGCCAGCMGCCGCGGTAA-3′), as previously described^3^.

### Microbiota analysis by 16 S rRNA gene sequencing

16 S rRNA gene amplification and sequencing were done using the Illumina MiSeq technology following the protocol of Earth Microbiome Project with their modifications to the MOBIO PowerSoil DNA Isolation Kit procedure for extracting DNA (www.earthmicrobiome.org/emp-standard-protocols). Bulk DNA were extracted from frozen extruded feces using a PowerSoil-htp kit from MoBio Laboratories (Carlsbad, California, USA) with mechanical disruption (bead-beating). The 16 S rRNA genes, region V4, were PCR amplified from each sample using a composite forward primer and a reverse primer containing a unique 12-base barcode, designed using the Golay error-correcting scheme, which was used to tag PCR products from respective samples^48^. We used the forward primer 515 F 5′-*AATGATACGGCGACCACCGAGATCTACAC*TATGGTAATT*GT*GTGCCAGCMGCCGCGGT AA-3′: the italicized sequence is the 5′ Illumina adapter B, the bold sequence is the primer pad, the italicized and bold sequence is the primer linker and the underlined sequence is the conserved bacterial primer 515 F. The reverse primer 806 R used was 5′-*CAAGCAGAAGACGGCATACGAGAT*XXXXXXXXXXXXAGTCAGTCAG*CC*GGACTACHVGGGTWTCTAAT-3′: the italicized sequence is the 3′ reverse complement sequence of Illumina adapter, the 12 × sequence is the golay barcode, the bold sequence is the primer pad, the italicized and bold sequence is the primer linker and the underlined sequence is the conserved bacterial primer 806 R. PCR reactions consisted of Hot Master PCR mix (Five Prime), 0.2 μm of each primer, 10–100 ng template, and reaction conditions were 3 min at 95 °C, followed by 30 cycles of 45 s at 95 °C, 60 s at 50 °C, and 90 s at 72 °C on a Biorad thermocycler. Four independent PCRs were performed for each sample, combined, purified with Ampure magnetic purification beads (Agencourt), and products were visualized by gel electrophoresis. Products were then quantified (BIOTEK Fluorescence Spectrophotometer) using Quant-iT PicoGreen dsDNA assay. A master DNA pool was generated from the purified products in equimolar ratios. The pooled products were quantified using Quant-iT PicoGreen dsDNA assay and then sequenced using an Illumina MiSeq sequencer (paired-end reads, 2 × 250 bp) at Cornell University, Ithaca.

### 16 S rRNA gene sequence analysis

Forward and reverse Illumina reads were joined using the fastq-join method^49,50^, sequences were demultiplexed, quality filtered using Quantitative Insights Into Microbial Ecology (QIIME, version 1.8.0) software package^51^. QIIME default parameters were used for quality filtering (reads truncated at first low-quality base and excluded if: (1) there were more than three consecutive low-quality base calls (2), < 75% of read length was consecutive high-quality base calls (3), at least one uncalled base was present (4), > 1.5 errors were present in the barcode (5), any Phred qualities were below 20, or (6) the length was < 75 bases). Sequences were clustered to operational taxonomic units (OTUs) using UCLUST algorithm^52^ with a 97% threshold of pairwise identity (without the creation of new clusters with sequences that do not match the reference sequences), and taxonomically classified using the Greengenes reference database 13_8^53^. A single representative sequence for each OTU was aligned and a phylogenetic tree was built using FastTree^54^. The phylogenetic tree was used for computing the weighted and unweighted UniFrac distances between samples^18,55^, rarefaction were performed and used to compare abundances of OTUs across samples. Principal coordinates analysis plots were used to assess the variation between experimental group (beta diversity). Alpha diversity curves were determined for all samples using the chao1 index. LEfSe (LDA Effect Size) was used to investigate bacterial members that drive differences between groups^56^. Unprocessed sequencing data are deposited in the European Nucleotide Archive under accession number PRJEB35012 (http://www.ebi.ac.uk/ena/data/view/PRJEB35012).

### Sample preparation for the identification of IgA-coated bacteria

IgA-coated bacteria were isolated and sequenced as previously described^19,20^. In brief, frozen fecal samples were thoroughly homogenized in PBS to a final concentration of 100 mg/mL. Fecal suspensions were filtered through a 40-μm sterile nylon mesh, then centrifuged at 50 × *g*, for 15 min at 4 °C. 100 μL of supernatant was then washed twice with 1 mL of staining buffer (PBS containing 1% (w/v) BSA) and centrifuged at 50 × *g*, for 15 min at 4 °C. Resulting bacterial pellets were resuspended in 100 μl blocking buffer (staining buffer containing 20% Normal Rat Serum) and incubated for 20 min on ice before being stained with 100 μl of staining buffer containing PE-conjugated Anti-Mouse IgA (1:12.5; eBioscience, 12–4204–82) for 30 min on ice. Following three washes with staining buffer, pellets were resuspended in 200 μL of 0.9%NaCl/0.1 m HEPES buffer (pH 7.2) containing a 1:4000 dilution of SytoBC (Invitrogen, S34855). Data acquisition was performed on a Sony Cell Sorter SH800Z. Samples were gated on appropriate SSC-A/FSC-A gates prior to being selected for SytoBC^+^ events. For each sample, 100,000 events were collected from the IgA^−^ and IgA^+^ population into sterile tubes. Each fraction was stored at − 20 °C prior to DNA extraction and sequencing of bacterial 16 S rRNA genes, as described above.

### Hematoxylin & eosin staining and histopathologic analysis

Following euthanasia, colons (proximal colon, 2 first cm from the cecum) were placed in methanol-Carnoy’s fixative solution (60% methanol, 30% chloroform, 10% glacial acetic acid) for a minimum of 3 h at room temperature. Tissues were then washed in methanol 2 × 30 min, ethanol 2 × 15 min, ethanol/xylene (1:1) 15 min, and xylene 2 × 15 min, followed by embedding in paraffin with a vertical orientation. Tissues were sectioned at 5-μm thickness and stained with hematoxylin & eosin (H&E) using standard protocols. H&E stained slides were assigned four scores based on the degree of epithelial damage and inflammatory infiltrate in the mucosa, submucosa, and muscularis/serosa, as previously described^57^. A slight modification was made to this scoring system, as we previously reported:^46^ each of the four scores was multiplied by 1 if the change was focal, 2 if it was patchy and 3 if it was diffuse. The four individual scores per colon were added, resulting in a total scoring range of 0–36 per mouse. Representative images were selected.

### Immunostaining of mucins and localization of bacteria by FISH

Mucus immunostaining was paired with fluorescent in situ hybridization (FISH), as previously described^58^, in order to analyze bacteria localization at the surface of the intestinal mucosa^3,9^. In brief, colonic tissues (proximal colon, 2nd cm from the cecum, without fecal material) were placed in methanol-Carnoy’s fixative solution (60% methanol, 30% chloroform, 10% glacial acetic acid) for a minimum of 3 h at room temperature. Tissues were then washed in methanol 2 × 30 min, ethanol 2 × 15 min, ethanol/xylene (1:1) 15 min, and xylene 2 × 15 min, followed by embedding in Paraffin with a vertical orientation. Five μm sections were performed and dewax by preheating at 60 °C for 10 min, followed by xylene 60 °C for 10 min, xylene for 10 min and 99.5% ethanol for 10 min Hybridization step was performed at 50 °C overnight with EUB338 probe (5′-GCTGCCTCCCGTAGGAGT-3′, with a 5′ labeling using Alexa 647) diluted to a final concentration of 10 μg/mL in hybridization buffer (20 mm Tris–HCl, pH 7.4, 0.9 m NaCl, 0.1% SDS, 20% formamide). After washing 10 min in wash buffer (20 mm Tris–HCl, pH 7.4, 0.9 m NaCl) and 3 × 10 min formamide in PBS, PAP pen (Sigma-Aldrich, Z377821) was used to mark around the section and block solution (5% fetal bovine serum in PBS) was added for 30 min at 4 °C. Mucin-2 primary antibody (rabbit H-300; Santa Cruz Biotechnology, sc-15334) was diluted 1:1500 in block solution and apply overnight at 4 °C. After washing 3 × 10 min in PBS, block solution containing anti-rabbit Alexa 488 secondary antibody diluted 1:1500, Phalloidin-Tetramethylrhodamine B isothiocyanate (Sigma-Aldrich, P1951) at 1 μg/mL and Hoechst 33258 (Sigma-Aldrich, 94403) at 10 μg/mL was applied to the section for 2 h. After washing 3 × 10 min in PBS slides were mounted using Prolong anti-fade mounting media (ThermoLife Technologies, P10144). Observations were performed with a Zeiss LSM 700 confocal microscope with software Zen 2011 version 7.1. This software was used to determine the distance between bacteria and epithelial cell monolayer, as previously described^14^. In brief, for each animal, two high-powered fields (HPF) were arbitrarily selected with the following inclusion criteria: (1) the presence of stained bacteria, (2) the presence of a clear and delimitated mucosal layer, and (3) the presence of an intact mucus layer. For each HPF, the distance between the five closest bacteria and the epithelium was determined. Thus, each bacterial–epithelial distance indicated by a point in the figures is, in fact, the average distance of 10 bacteria–epithelial distances.

### DSS-induced acute-colitis

Mice were administered DSS (MP Biomedicals, 0216011001) at 2.5% in drinking water ad libitum for 10 days, as previously described^46^. Control mice were given water only. During this period, mice were weighed every other day. After 10 days, mice were killed by CO_2_ euthanasia and samples were collected as described above.

### Human subjects

All work involving human subjects used a previously existing stool collection, described in ref. ^23^, which was generated under approval by Cornell University (Ithaca NY); IRB Protocol ID 1108002388. As previously described^23^, fecal samples were collected at home by participants in the United Kingdom Adult Twin Registry (TwinsUK)^59^ in 15 ml conical tubes and refrigerated for 1–2 days prior to the participants’ annual clinical visits at King’s College London (KCL). Upon arrival at KCL, the samples were stored at −80 °C and shipped by courier on dry ice to Cornell University, where they were stored at −80 °C until processing.

### Statistical analysis

Significance was determined using *t* test or one-way ANOVA with Bonferroni multiple comparisons test (GraphPad Prism software, version 8.2.0). Differences were noted as significant *p ≤ 0.05 **p ≤ 0.01 ***p ≤ 0.001 ****p ≤ 0.0001 for *t* test and ^#^*p* ≤ 0.05 ^##^*p* ≤ 0.01 ^###^*p* ≤ 0.001 ^####^*p* ≤ 0.0001 for one-way ANOVA. For clustering analyzing on principal coordinate plots, categories were compared and statistical significance of clustering were determined via Permanova.

### Reporting summary

Further information on research design is available in the Nature Research Reporting Summary linked to this article.

## Supplementary information

Supplementary Information

Reporting Summary

## Data Availability

Unprocessed sequencing data are deposited in the European Nucleotide Archive under accession number PRJEB35012. Other data generated or analyzed during this study are included in this published article and its Supplementary Information files.

## Source data

Source data
