# Supplementary material for: Flagellin-elicited adaptive immunity suppresses flagellated microbiota and vaccinates against chronic inflammatory diseases
Source: Nat Commun. 2019 Dec 11;10:5650. doi: 10.1038/s41467-019-13538-y (PMC6906489; doi:10.1038/s41467-019-13538-y)
Supplement: Supplementary file 1 — Supplementary Information [file 41467_2019_13538_MOESM1_ESM.pdf]

Flagellin-elicited adaptive immunity suppresses flagellated microbiota and vaccinates against chronic inflammatory diseases

Tran et al.

Supplementary Information

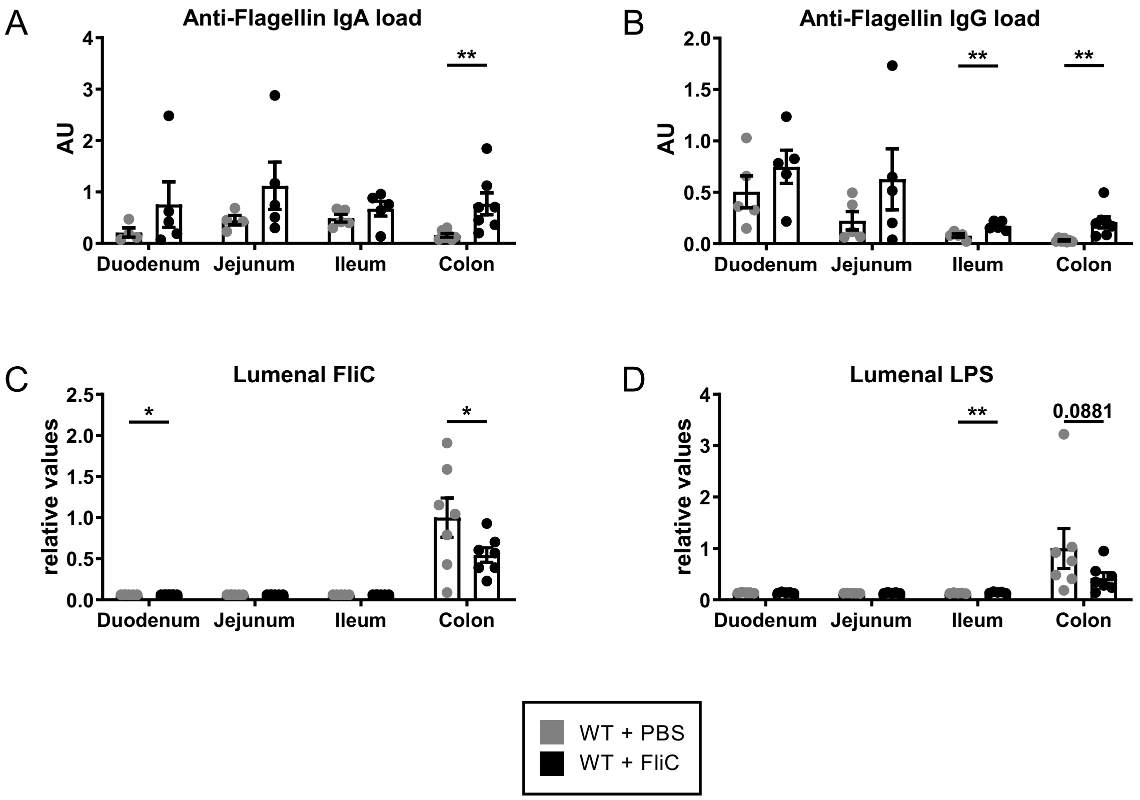

Supplemental Figure 1. **Localization of the adaptive immune response induced by flagellin immunization.** C57BL/6J Wild Type mice were purchased from The Jackson Laboratory and housed for two weeks before procedure in order to favor microbiota stabilization. Next, flagellin (10 µg per mouse) was administered by intraperitoneal injections weekly for 9 weeks, while control mice received vehicle (PBS). Following euthanasia, intestinal contents were collected and analyzed for (**A**) anti-flagellin IgA and (**B**) anti-flagellin IgG using ELISA kits. Additionally, intestinal contents were analyzed for (**C**) fecal flagellin and (**D**) fecal LPS using HEK 293 cells expressing mTLR5 or mTLR4 measuring bioactive flagellin and lipopolysaccharide, respectively. Data are the means +/- S.E.M.. Significance was determined using *t*-test (*$p \le 0.05$ **$p \le 0.01$). (N=4-5 mice). Source data are provided as a Source Data file.

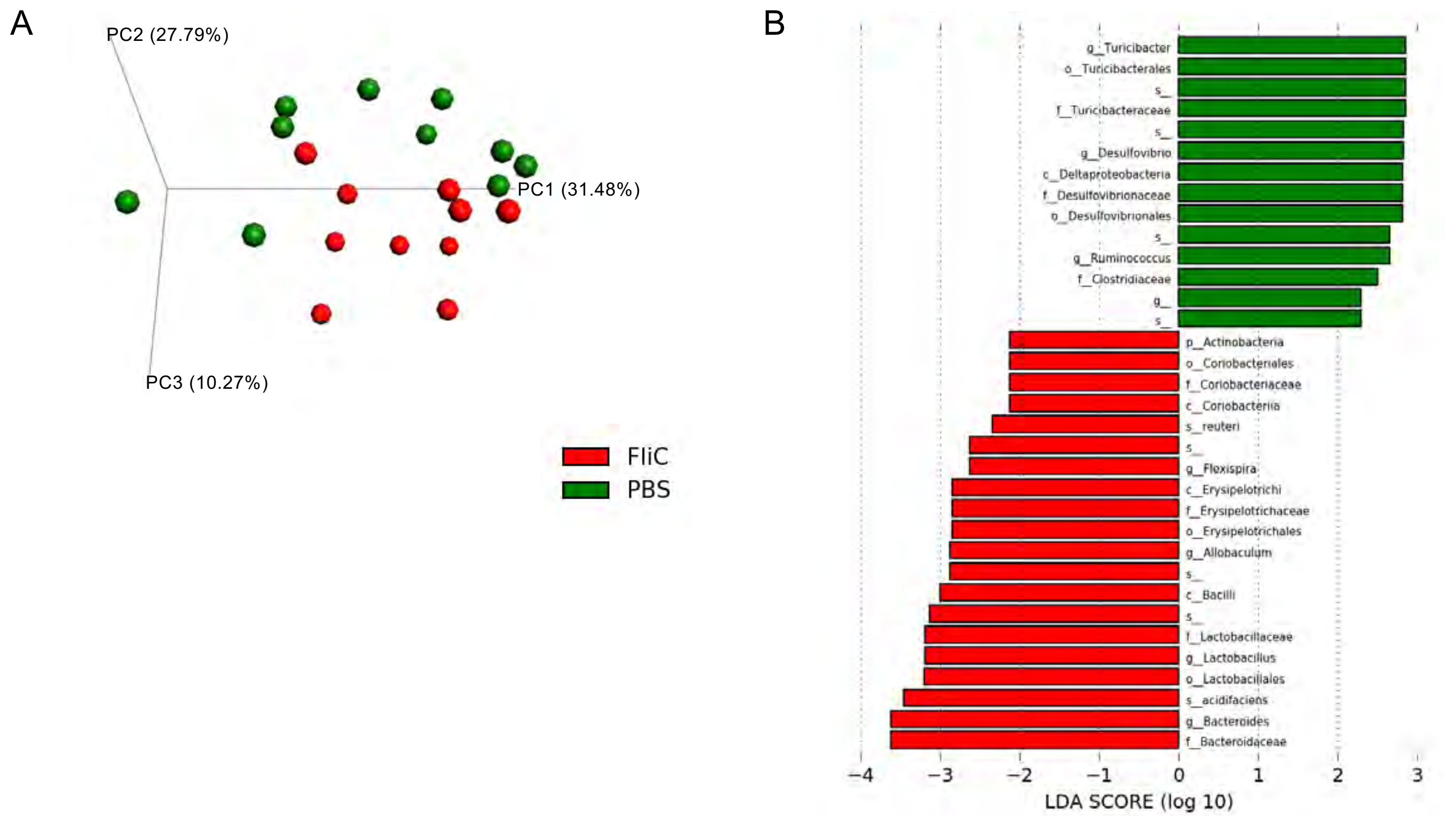

Supplemental Figure 2: Microbiota composition analysis in flagellin immunized vs. non-immunized mice. 4-week old C57BL/6J mice were immunized with flagellin (10 µg per mouse) by intraperitoneal injections weekly for 9 weeks, while control mice received vehicle (PBS). Fecal microbiota composition was analyzed using Illumina sequencing of the V4 region of 16S rRNA genes. (**A**) Principal coordinates analysis (PCoA) of the weighted UniFrac distance matrix at day 56 (post-stabilization, post-immunization). (**B**) LEfSe analysis at day 56 reveals multiple bacterial groups altered by flagellin immunization compared to PBS treated mice.

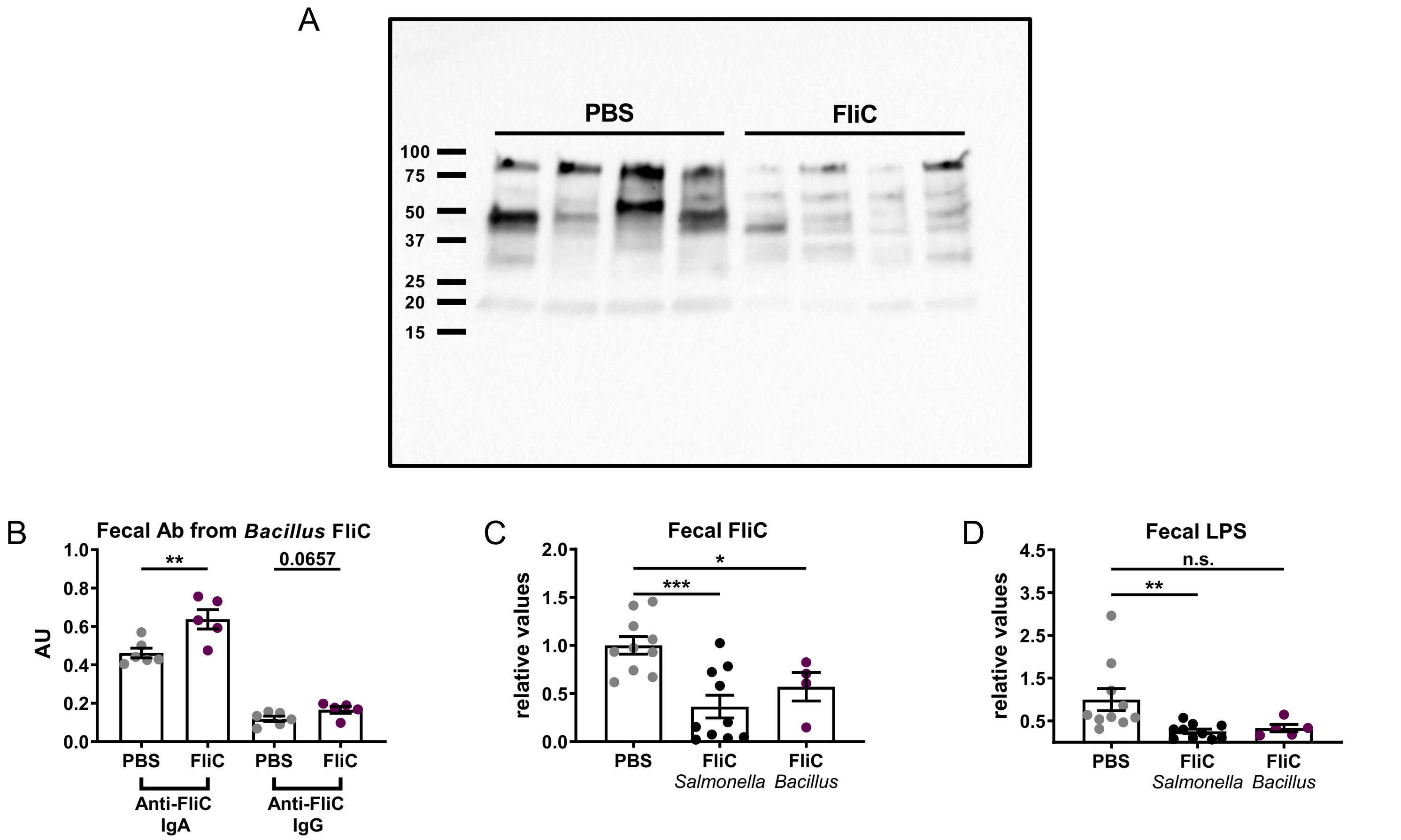

Supplemental Figure 3: Functional consequences of flagellin immunization on the intestinal microbiota. 4-week old C57BL/6J mice were immunized with *Salmonella*-derived or *Bacillus*-derived flagellin (10 µg per mouse) by intraperitoneal injections weekly for 9 weeks, while control mice received vehicle (PBS). (**A**) Western blot of fecal samples from PBS or *Salmonella*-derived flagellin treated mice using an anti-flagellin primary antibody. (**B**) Fecal anti-flagellin IgA and IgG quantification in mice treated with vehicle or *Bacillus*-derived flagellin. (**C**) Fecal flagellin and (**D**) fecal LPS were analyzed using HEK 293 cells expressing mTLR5 or mTLR4 measuring bioactive flagellin and lipopolysaccharide, respectively. Data are the means +/- S.E.M.. Significance was determined using *t*-test (*$p \leq 0.05$ **$p \leq 0.01$ ***$p \leq 0.001$, n.s. indicates non-significant). (N=4-5 mice). Source data are provided as a Source Data file.

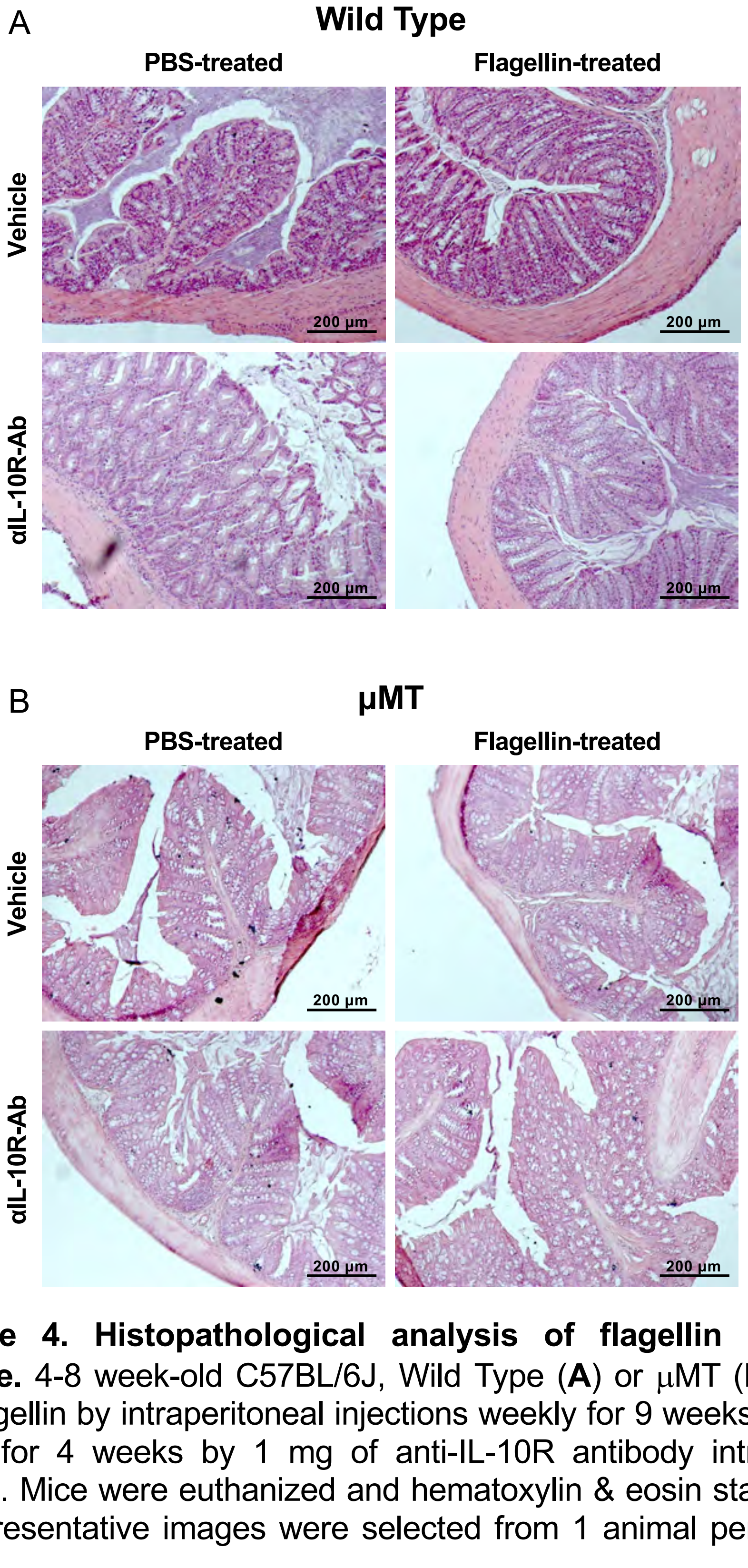

Supplemental Figure 4. Histopathological analysis of flagellin immunized and non-immunized μMT mice. 4-8 week-old C57BL/6J, Wild Type (**A**) or μMT (**B**), mice received either vehicle or 10 µg of flagellin by intraperitoneal injections weekly for 9 weeks. Subsequently, animals were treated weekly for 4 weeks by 1 mg of anti-IL-10R antibody intraperitoneally to induce intestinal inflammation. Mice were euthanized and hematoxylin & eosin staining was performed on colonic sections. Representative images were selected from 1 animal per cage of (**A**) Wild Type and (**B**) μMT mice.

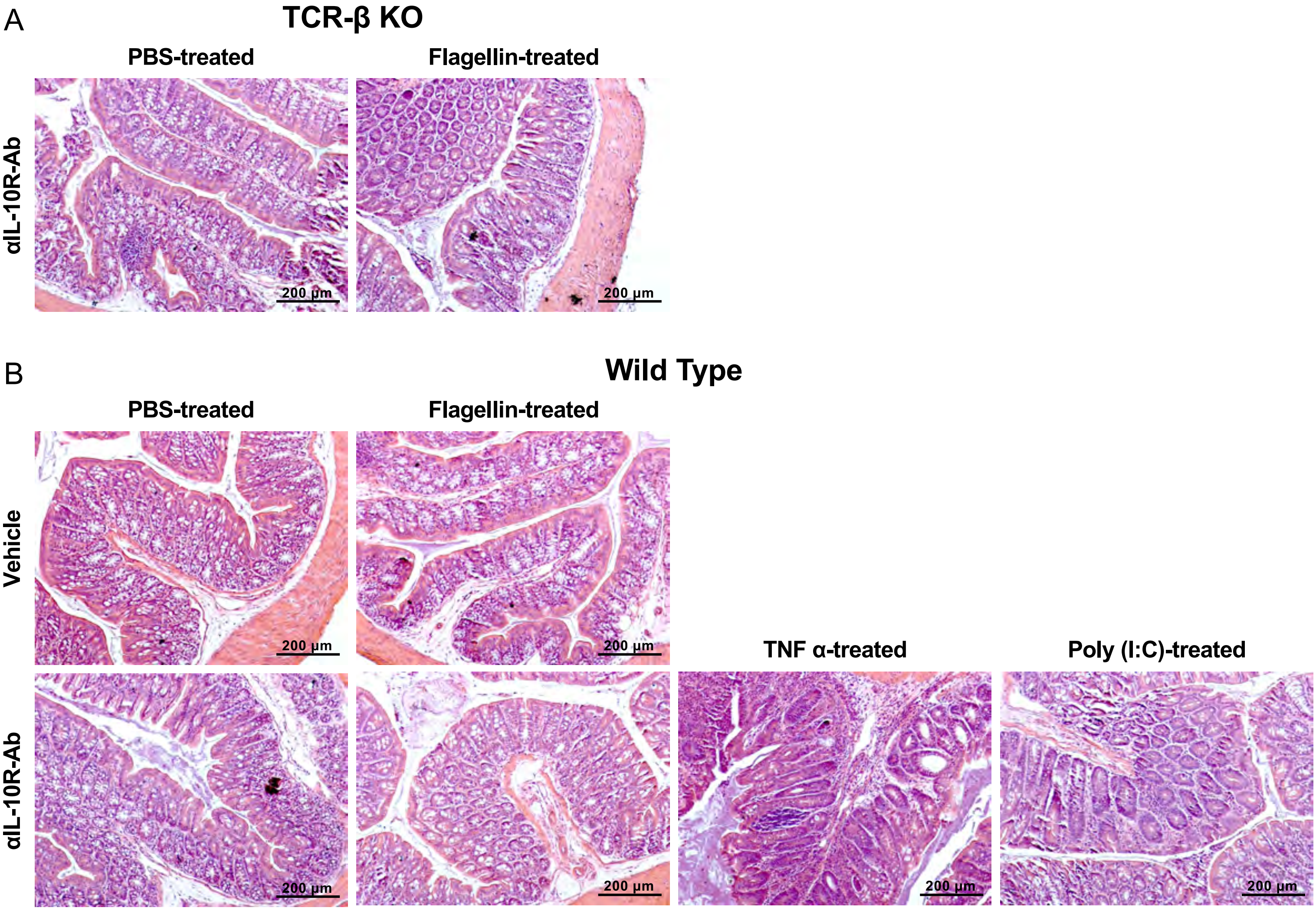

Supplemental Figure 5. Histopathological analysis of flagellin immunized and non-immunized TCRβ KO mice. 4-week old C57BL/6J TCRβ KO (**A**) and Wild Type (**B**) mice were purchased from The Jackson Laboratory and housed for two weeks before procedure in order to favor microbiota stabilization. Subsequently, mice were treated with either flagellin (10 µg per mouse), TNF-α (50 µg/kg body weight), or Poly (I:C) (10 µg/kg body weight) *via* intraperitoneal injections weekly for 9 weeks, while control mice received vehicle (PBS). Animals were then treated weekly for 4 weeks by 1 mg of anti-IL-10R antibody intraperitoneally to induce intestinal inflammation. Mice were euthanized and hematoxylin & eosin staining was performed on colonic sections. Representative images were selected from 1 animal per cage of (**A**) TCRβ KO and (**B**) Wild Type mice.

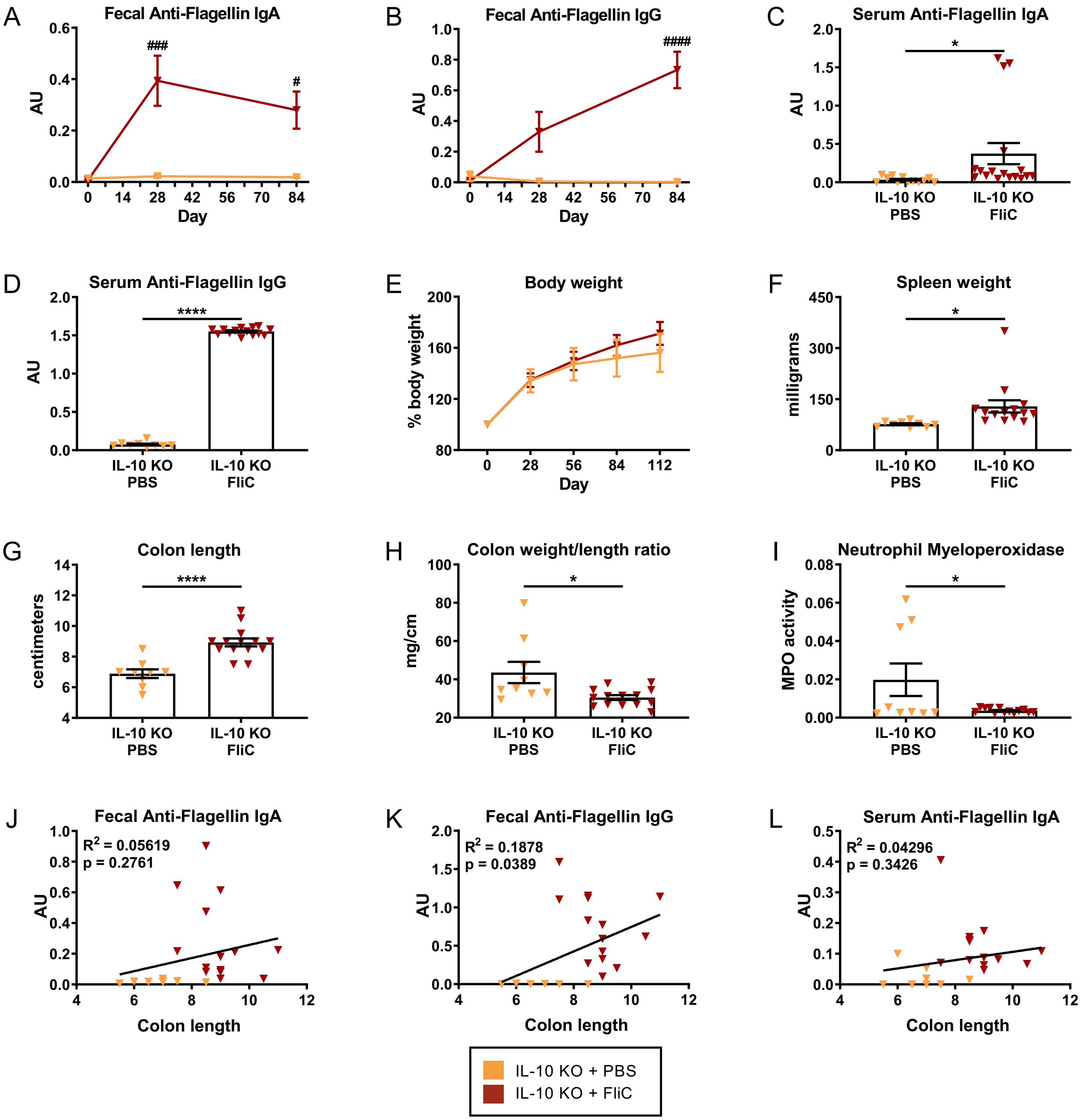

Supplemental Figure 6: Flagellin immunization protects against spontaneous colitis in IL-10 KO mice. 6-8 week-old, IL-10 KO mice received either vehicle or 10 µg of flagellin by intraperitoneal injections weekly for 17 weeks before euthanasia. (**A-B**) Fecal anti-flagellin IgA and IgG. (**C-D**) Day 112 serum anti-flagellin IgA and IgG. (**E**) Body weights were measured weekly and expressed as relative values, day 0 being define as 100%. (**F**) Spleen weight. (**G**) Colon length. (**H**) Colon weight/length ratio. (**I**) Colonic myeloperoxidase levels. (**J-L**) Correlation of colon length from IL-10 KO mice and Day 112 fecal IgA and IgG, and Day 112 serum IgA. Data are the means +/- S.E.M.. Significance was determined using linear regression analysis (for **J-L**, p values shown), *t*-test (**p*≤0.05 *****p*≤0.0001), or using one-way ANOVA corrected for multiple comparisons with a Bonferroni test (#*p*≤0.05 ### *p*≤0.001 #### *p*≤0.0001). (*N*=9-13). Source data are provided as a Source Data file.

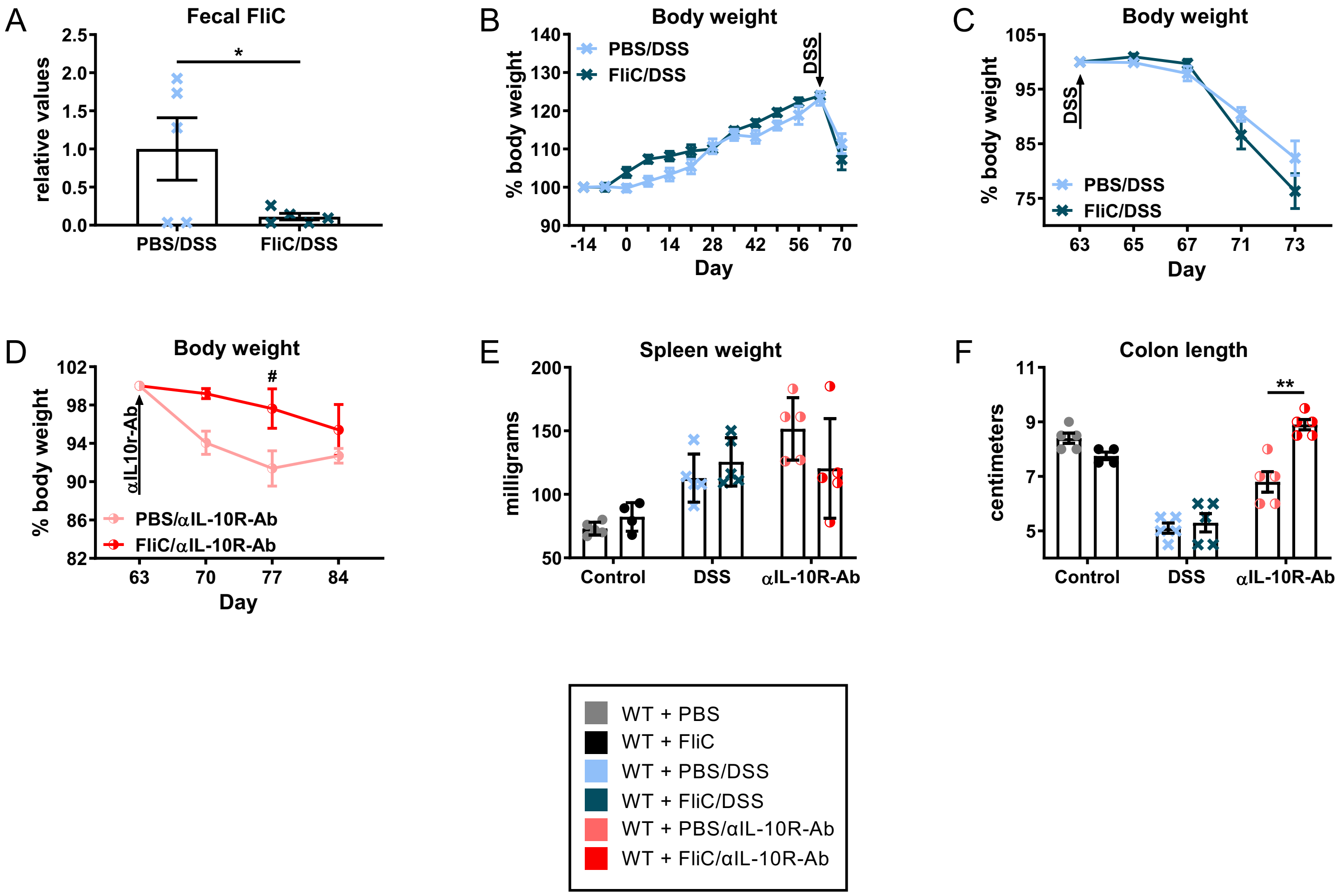

Supplemental Figure 7: Flagellin shows repeated protection against anti-IL-10R treatment but does not confer protection against DSS induced acute intestinal inflammation. 8-week old, Wild Type C57BL/6J mice were purchased from The Jackson Laboratory and housed for two weeks before procedure in order to favor microbiota stabilization. Subsequently, flagellin (10 µg per mouse) was administered by intraperitoneal injections weekly for 9 weeks, while control mice received vehicle (PBS). Subsequently, colitis was induced by either 4 weekly intraperitoneal injections of 1 mg anti-IL-10R antibody or by treatment with DSS diluted in the drinking water (2.5%). (**A**) Fecal flagellin quantified using HEK 293 cells expressing mTLR5. Body weights were measured weekly and expressed as relative values, (**B**) day -14 (pre-immunization) or (**C-D**) day 63 (post-immunization, pre colitis induction) being define as 100%. (**E**) Spleen weight. (**F**) Colon length. Data are the means +/- S.E.M.. Significance was determined using *t*-test (**p*≤0.05 ***p*≤0.01) or using one-way ANOVA corrected for multiple comparisons with a Bonferroni test (#*p*≤0.05). (*N*=4-5). Source data are provided as a Source Data file.
