# Supplementary material for: Flagellin-elicited adaptive immunity suppresses flagellated microbiota and vaccinates against chronic inflammatory diseases
Source: Nat Commun. 2019 Dec 11;10:5650. doi: 10.1038/s41467-019-13538-y (PMC6906489; doi:10.1038/s41467-019-13538-y)
Supplement: Supplementary file 2 — Reporting Summary [file 41467_2019_13538_MOESM2_ESM.pdf]

nature research

Corresponding author(s): Benoit Chassaing

Last updated by author(s): 2019/10/21

Reporting Summary

Nature Research wishes to improve the reproducibility of the work that we publish. This form provides structure for consistency and transparency in reporting. For further information on Nature Research policies, see Authors & Referees and the Editorial Policy Checklist.

Statistics

For all statistical analyses, confirm that the following items are present in the figure legend, table legend, main text, or Methods section.

n/a	Confirmed	
☐	☒	The exact sample size (*n*) for each experimental group/condition, given as a discrete number and unit of measurement
☒	☐	A statement on whether measurements were taken from distinct samples or whether the same sample was measured repeatedly
☐	☒	The statistical test(s) used AND whether they are one- or two-sided<br>*Only common tests should be described solely by name; describe more complex techniques in the Methods section.*
☒	☐	A description of all covariates tested
☐	☒	A description of any assumptions or corrections, such as tests of normality and adjustment for multiple comparisons
☐	☒	A full description of the statistical parameters including central tendency (e.g. means) or other basic estimates (e.g. regression coefficient) AND variation (e.g. standard deviation) or associated estimates of uncertainty (e.g. confidence intervals)
☒	☐	For null hypothesis testing, the test statistic (e.g. *F*, *t*, *r*) with confidence intervals, effect sizes, degrees of freedom and *P* value noted<br>*Give P values as exact values whenever suitable.*
☒	☐	For Bayesian analysis, information on the choice of priors and Markov chain Monte Carlo settings
☒	☐	For hierarchical and complex designs, identification of the appropriate level for tests and full reporting of outcomes
☒	☐	Estimates of effect sizes (e.g. Cohen's *d*, Pearson's *r*), indicating how they were calculated

Our web collection on statistics for biologists contains articles on many of the points above.

Software and code

Policy information about availability of computer code

Data collection	N.A.
Data analysis	Quantitative Insights Into Microbial Ecology (QIIME, version 1.8.0)

For manuscripts utilizing custom algorithms or software that are central to the research but not yet described in published literature, software must be made available to editors/reviewers. We strongly encourage code deposition in a community repository (e.g. GitHub). See the Nature Research guidelines for submitting code & software for further information.

Data

Policy information about availability of data

All manuscripts must include a data availability statement. This statement should provide the following information, where applicable:

- Accession codes, unique identifiers, or web links for publicly available datasets
- A list of figures that have associated raw data
- A description of any restrictions on data availability

Unprocessed sequencing data are deposited in the European Nucleotide Archive under accession number PRJEB35012 (http://www.ebi.ac.uk/ena/data/view/PRJEB35012).

Field-specific reporting

Please select the one below that is the best fit for your research. If you are not sure, read the appropriate sections before making your selection.

☒ Life sciences ☐ Behavioural & social sciences ☐ Ecological, evolutionary & environmental sciences

For a reference copy of the document with all sections, see nature.com/documents/nr-reporting-summary-flat.pdf

Life sciences study design

All studies must disclose on these points even when the disclosure is negative.

Sample size	N.A.
Data exclusions	No data were excluded from the analysis.
Replication	All replicates are presented.
Randomization	N.A.
Blinding	Blinding was used for histopahological scoring as well as for the measure of microbiota / epithelium distance.

Reporting for specific materials, systems and methods

We require information from authors about some types of materials, experimental systems and methods used in many studies. Here, indicate whether each material, system or method listed is relevant to your study. If you are not sure if a list item applies to your research, read the appropriate section before selecting a response.

Materials & experimental systems

n/a	Involved in the study	
☐	☒	Antibodies
☐	☒	Eukaryotic cell lines
☒	☐	Palaeontology
☐	☒	Animals and other organisms
☐	☒	Human research participants
☒	☐	Clinical data

Methods

n/a	Involved in the study	
☒	☐	ChIP-seq
☐	☒	Flow cytometry
☒	☐	MRI-based neuroimaging

Antibodies

Antibodies used	rabbit H-300; Santa Cruz Biotechnology, sc-15334<br>anti-flagellin Invivogen, mabg-flast
Validation	N.A.

Eukaryotic cell lines

Policy information about cell lines

Cell line source(s)	Invivogen
Authentication	HEK-TLR5 HEK-TLR4
Mycoplasma contamination	N.A.
Commonly misidentified lines<br>(See ICLAC register)	N.A.

Animals and other organisms

Policy information about studies involving animals; ARRIVE guidelines recommended for reporting animal research

Laboratory animals	Mus musculus
Wild animals	*Provide details on animals observed in or captured in the field; report species, sex and age where possible. Describe how animals were caught and transported and what happened to captive animals after the study (if killed, explain why and describe method; if released, say where and when) OR state that the study did not involve wild animals.*
Field-collected samples	*For laboratory work with field-collected samples, describe all relevant parameters such as housing, maintenance, temperature, photoperiod and end-of-experiment protocol OR state that the study did not involve samples collected from the field.*

Ethics oversight	Animals were maintained at Georgia State University, Atlanta, Georgia, USA under institutionally approved protocols (IACUC # A14033 and A18006).

Note that full information on the approval of the study protocol must also be provided in the manuscript.

Human research participants

Policy information about studies involving human research participants

Population characteristics	All work involving human subjects used a previously existing stool collection, described in 23, which was generated under approval by Cornell University (Ithaca NY); IRB Protocol ID 1108002388.
Recruitment	All work involving human subjects used a previously existing stool collection, described in 23, which was generated under approval by Cornell University (Ithaca NY); IRB Protocol ID 1108002388.
Ethics oversight	All work involving human subjects used a previously existing stool collection, described in 23, which was generated under approval by Cornell University (Ithaca NY); IRB Protocol ID 1108002388.

Note that full information on the approval of the study protocol must also be provided in the manuscript.

Flow Cytometry

Plots

Confirm that:

☒ The axis labels state the marker and fluorochrome used (e.g. CD4-FITC).

☒ The axis scales are clearly visible. Include numbers along axes only for bottom left plot of group (a 'group' is an analysis of identical markers).

☒ All plots are contour plots with outliers or pseudocolor plots.

☒ A numerical value for number of cells or percentage (with statistics) is provided.

Methodology

Sample preparation	IgA-coated bacteria were isolated and sequenced as previously described 19, 20. Briefly, frozen fecal samples were thoroughly homogenized in PBS to a final concentration of 100 mg/mL. Fecal suspensions were filtered through a 40 µm sterile nylon mesh, then centrifuged at 50 x g, for 15 minutes at 4°C. 100 ?L of supernatant was then washed twice with 1 mL of staining buffer (PBS containing 1% (w/v) BSA) and centrifuged at 50 x g, for 15 minutes at 4°C. Resulting bacterial pellets were resuspended in 100 µl blocking buffer (staining buffer containing 20% Normal Rat Serum) and incubated for 20 minutes on ice before being stained with 100 µl of staining buffer containing PE-conjugated Anti-Mouse IgA (1:12.5; eBioscience, 12-4204-82) for 30 minutes on ice. Following three washes with staining buffer, pellets were resuspended in 200 µL of 0.9%NaCl/0.1 M HEPES buffer (pH 7.2) containing a 1:4000 dilution of SytoBC (Invitrogen, S34855). Data acquisition was performed on a Sony Cell Sorter SH800Z. Samples were gated on appropriate SSC-A/FSC-A gates prior to being selected for SytoBC+ events. For each sample, 100,000 events were collected from the IgA- and IgA+ population into sterile tubes. Each fraction was stored at -20°C prior to DNA extraction and sequencing of bacterial 16S rRNA genes, as described above.
Instrument	Sony Cell Sorter SH800Z
Software	FlowJo
Cell population abundance	N.A.
Gating strategy	N.A.

☒ Tick this box to confirm that a figure exemplifying the gating strategy is provided in the Supplementary Information.
